# Integrative effects of saffron and physical activity on endurance performance, quality of life, cognitive, emotional, and metabolic outcomes in age-related and neurodegenerative diseases

**DOI:** 10.3389/fnut.2025.1698135

**Published:** 2025-11-27

**Authors:** Lingyun Li, Kehai Wang

**Affiliations:** 1 Physical Education College, Shandong University of Finance and Economics, Jinan, Shandong, China; 2 Department of Physical Education, Shandong University of Traditional Chinese Medicine, Jinan, Shandong, China

**Keywords:** saffron, exercise, aging, Alzheimer's disease, Parkinson's disease

## Abstract

Age-related diseases, including cardiovascular disorders, type 2 diabetes, neurodegenerative conditions such as Alzheimer's and Parkinson's disease, and age-related eye diseases, represent leading causes of disability and mortality worldwide. Growing evidence highlights the therapeutic promise of non-pharmacological interventions, notably saffron (*Crocus sativus* L.) and structured exercise, both of which exert pleiotropic effects through antioxidant, anti-inflammatory, and neuroprotective pathways. In this review, we summarize current experimental and clinical data on saffron's bioactive compounds, crocin, crocetin, and safranal, and their capacity to modulate lipid metabolism, insulin sensitivity, mitochondrial function, and protein aggregation. Parallel findings from exercise research demonstrate improvements in cardiovascular function, glycemic control, neuroplasticity, and ocular health. Importantly, emerging studies reveal synergistic benefits when saffron supplementation is combined with physical activity, resulting in amplified improvements in vascular remodeling, glycemic regulation, neurotrophic signaling, and behavioral outcomes. These complementary interventions target shared molecular pathways, including PI3K/Akt/mTOR signaling, SIRT1–PGC-1α activation, Nrf2-mediated antioxidant defense, and modulation of inflammatory cytokines. Taken together, saffron and exercise represent safe, accessible, and multi-target strategies that may delay or attenuate the progression of aging-related diseases. Future large-scale, long-term clinical trials are warranted to establish optimal protocols and to integrate these interventions into preventive and therapeutic frameworks for healthy aging.

## 1 Introduction

The global demographic shift toward an increasingly older population has become one of the most pressing public health concerns of the current era. Advances in medicine, technology, and socioeconomic development have extended life expectancy, but these gains are accompanied by a steep rise in age-associated diseases (1). By 2030, one in six individuals worldwide will be aged 60 years or older, and by 2050, the number of people in this age group is expected to reach 2.1 billion, with the majority living in low- and middle-income countries. The extension of human life span is not necessarily paralleled by a similar expansion in health span. Many individuals spend their final decades coping with chronic, debilitating disorders that severely impact independence and quality of life (2). Neurological decline, cardiometabolic dysfunction, and psychiatric disturbances rank among the leading contributors to morbidity in older adults, creating both medical and socioeconomic burdens for families and healthcare systems (3).

Age-related diseases encompass a broad spectrum of conditions, including neurodegenerative disorders such as Alzheimer's disease (AD), Parkinson's disease (PD), Huntington's disease (HD), multiple sclerosis (MS), and amyotrophic lateral sclerosis (ALS), as well as cardiovascular disease (CVD), type 2 diabetes (T2D), metabolic syndrome, and certain cancers (4, 5). These conditions are driven by a complex interplay of genetic, molecular, and environmental factors, with aging itself serving as the strongest risk factor (6). Biological aging is marked by cumulative damage at the cellular and molecular levels, involving oxidative stress, mitochondrial dysfunction, chronic inflammation, impaired proteostasis, and dysregulated signaling networks (7). These alterations ultimately compromise physiological resilience, leading to cognitive decline, emotional instability, impaired mobility, and increased vulnerability to chronic diseases.

Neurodegenerative and cardiometabolic disorders are leading contributors to age-related morbidity, characterized by progressive neuronal loss, impaired cognition and movement, metabolic dysregulation, and heightened vulnerability to conditions such as dementia, Parkinson's disease, type 2 diabetes, and cardiovascular disease (8). These conditions often coexist with sarcopenia, depression, and anxiety, creating a cycle of functional decline and diminished quality of life (9). Current pharmacological options provide only temporary symptomatic relief and are limited by side effects, underscoring the urgent need for safe, affordable, and multi-targeted strategies (10). Increasingly, nutraceuticals like saffron and lifestyle approaches, such as structured physical activity, are being explored as promising non-pharmacological interventions to address these interconnected challenges of aging.

Among natural products, *Crocus sativus L*., commonly known as saffron, stands out as a promising candidate. Saffron is a highly valued spice with a long history of use in traditional medicine across Persian, Mediterranean, and South Asian cultures (11). Modern scientific research has revealed that saffron and its active constituents, including crocin, crocetin, safranal, and picrocrocin, possess a wide range of pharmacological activities (12, 13). These compounds exhibit strong antioxidant capacity, modulate inflammatory signaling, regulate apoptosis and autophagy, and influence neurotransmitter systems, such as serotonin, dopamine, and glutamate (14). Through these mechanisms, saffron has been shown to protect neuronal cells, enhance cognitive performance, and alleviate mood disorders (15). Clinical trials suggest that saffron supplementation can improve symptoms of mild-to-moderate depression with fewer side effects compared to conventional antidepressants (16). In models of AD, saffron reduces amyloid-beta accumulation and improves memory performance, while in PD, it preserves dopaminergic neurons and mitigates motor dysfunction. Importantly, saffron's safety profile and minimal toxicity make it an attractive long-term intervention for older adults (17, 18).

Parallel to nutraceutical research, physical activity has long been recognized as a cornerstone of preventive medicine in aging. Regular exercise confers multidimensional benefits across neurological, psychological, and metabolic domains (19). Aerobic exercise enhances cardiovascular health, increases insulin sensitivity, and supports neurogenesis, while resistance training is particularly effective in combating sarcopenia and mobility disability (20–23). Exercise also stimulates the release of neurotrophic factors, such as brain-derived neurotrophic factor (BDNF), which promote synaptic plasticity, learning, and memory (24). Moreover, physical activity modulates the hypothalamic–pituitary–adrenal (HPA) axis, thereby reducing stress reactivity and improving mood stability (25). Evidence from large epidemiological studies shows that physically active individuals have a significantly lower risk of dementia, depression, cardiovascular events, and premature mortality (26).

Interestingly, both saffron and physical activity appear to converge on similar molecular pathways, though they act through distinct upstream triggers. For instance, both interventions enhance mitochondrial function, reduce oxidative stress, and attenuate chronic low-grade inflammation, all of which are central to the biology of aging (27, 28). Both also regulate neurotransmitter systems and promote neurotrophic signaling, supporting cognitive resilience (29, 30). Moreover, they complement each other: while exercise directly improves metabolic health and muscular function, saffron provides bioactive compounds that can cross the blood–brain barrier and modulate neurochemical balance (15, 31). Together, these interventions may reinforce each other, creating synergistic effects that are greater than either strategy alone.

Another important dimension is their combined potential to improve the quality of life. Older adults often struggle with fatigue, sleep disturbances, reduced motivation, and emotional instability, which can undermine adherence to exercise regimens (32). Saffron supplementation, by improving mood, sleep quality, and energy (33), may enhance the capacity and willingness of individuals to engage in physical activity. Conversely, exercise may optimize the body's utilization and metabolism of saffron's bioactives, potentially amplifying their efficacy. Such bidirectional reinforcement highlights the value of integrative approaches that bridge nutraceutical and lifestyle domains.

The urgency of developing such integrative strategies is underscored by global policy frameworks. The World Health Organization has declared 2021–2030 the “Decade of Healthy Ageing,” emphasizing the need for holistic, person-centered interventions that enable older adults to maintain functional ability and wellbeing. This initiative calls for the development of evidence-based, low-cost strategies that can be implemented across diverse cultural and socioeconomic contexts (34, 35). Saffron, with its long-standing cultural acceptance and safety, combined with universally applicable exercise programs, aligns well with these global goals.

The present review aims to synthesize current evidence on the individual and combined effects of saffron supplementation and physical activity in aging. We specifically highlight their roles in modulating cognitive, emotional, and metabolic outcomes, and delineate shared and distinct molecular pathways that underlie these effects. By mapping areas of convergence and synergy, this review proposes an integrative framework for non-pharmacological strategies to enhance quality of life and resilience in older adults. Ultimately, combining saffron with structured exercise may represent a safe, accessible, and culturally adaptable approach to promote healthy longevity and mitigate the burden of age-related diseases.

## 2 Bioactive compounds of saffron

**Figure 1:**
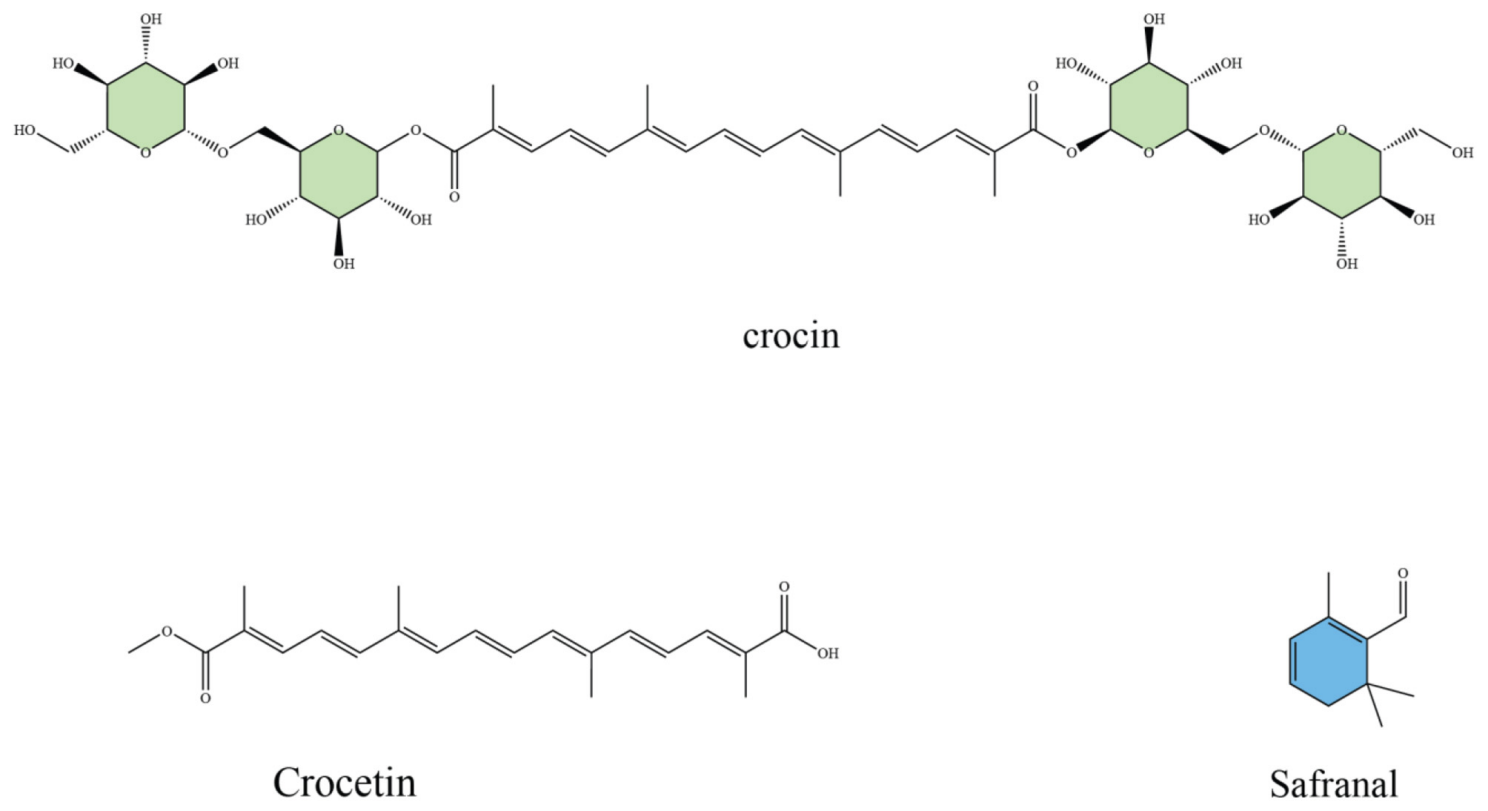
Chemical structures of major saffron bioactive constituents: crocin (a glycosylated carotenoid responsible for saffron's color and antioxidant activity), crocetin (an aglycone carotenoid with vascular and metabolic effects), and safranal (a monoterpene aldehyde contributing to aroma and neuroprotective properties).

Saffron (*Crocus sativus L*.) is considered one of the most chemically diverse medicinal plants, with over 160 bioactive molecules identified in its floral stigmas, tepals, and corms (36). Among these, four compounds, crocin, crocetin, picrocrocin, and safranal (Figure 1), are regarded as the main active constituents that account for its unique sensory properties and its wide spectrum of biological activities (37). Crocin represents the most abundant apocarotenoid in saffron, responsible for its characteristic deep red coloration and high solubility in water (38). Beyond its role as a natural dye, crocin acts as a potent antioxidant, efficiently scavenging reactive oxygen species (ROS) and protecting neuronal and cardiovascular tissues from oxidative injury (39, 40). Experimental studies further highlight its neuroprotective actions, including enhancement of memory performance, attenuation of β-amyloid–induced toxicity in Alzheimer's disease models, and improvement of dopaminergic survival in Parkinson's models (41, 42). Crocin has also been linked to antidepressant-like effects, partly through modulation of serotonin and dopamine signaling (43, 44).

Crocetin, the aglycone form of crocin, is a lipophilic carotenoid that contributes to saffron's orange–red hue. Although less abundant, crocetin exhibits important pharmacological effects such as suppression of lipid peroxidation, regulation of mitochondrial function, and anti-inflammatory activity via downregulation of pro-inflammatory cytokines (45, 46). Animal and human studies indicate that crocetin improves insulin sensitivity, lowers serum lipids, and protects vascular endothelium, suggesting a role in metabolic health and cardiovascular disease prevention (47–49).

Picrocrocin, a monoterpene glycoside, is the precursor of saffron's distinctive bitter flavor (50). While traditionally valued as a flavoring compound, picrocrocin has demonstrated bioactivity in experimental systems, including anti-proliferative effects on certain cancer cell lines and modulation of apoptotic pathways (51). Its breakdown during dehydration or storage leads to the formation of safranal, another major bioactive (52). Safranal, the main volatile aldehyde generated from picrocrocin, is primarily responsible for saffron's aroma (53). Despite being present in relatively low concentrations, safranal displays significant pharmacological effects. Studies have reported its strong antioxidant capacity, cytotoxicity against tumor cells, as well as antidepressant, anxiolytic, and anticonvulsant activities. Its role in neuronal protection has been attributed to both free radical scavenging and regulation of neurotransmitter systems (54).

Together, these compounds act through overlapping mechanisms, particularly antioxidant, anti-inflammatory, and anti-apoptotic pathways, that are central to aging-related disorders. In addition, they modulate neurotransmitter balance, support mitochondrial function, and regulate glucose and lipid metabolism, linking saffron not only to neuroprotection but also to cardiometabolic health. The unique combination of sensory attributes and pharmacological properties makes saffron an exceptional source of natural bioactives with wide therapeutic potential. Collectively, these molecular and physiological properties highlight saffron as a scientifically substantiated nutraceutical rather than merely a traditional remedy. Its key constituents, crocin, crocetin, safranal, and picrocrocin, act on convergent cellular pathways that regulate mitochondrial bioenergetics, neurotransmitter balance, and protein aggregation, providing a mechanistic justification for its antioxidant, anti-inflammatory, and neuroprotective efficacy. By bridging traditional ethnopharmacological knowledge with modern biochemical validation, saffron exemplifies how plant-derived bioactives can contribute to evidence-based preventive and therapeutic strategies in aging and neurodegenerative disease.

## 3 Saffron and age-related health

**Figure 2:**
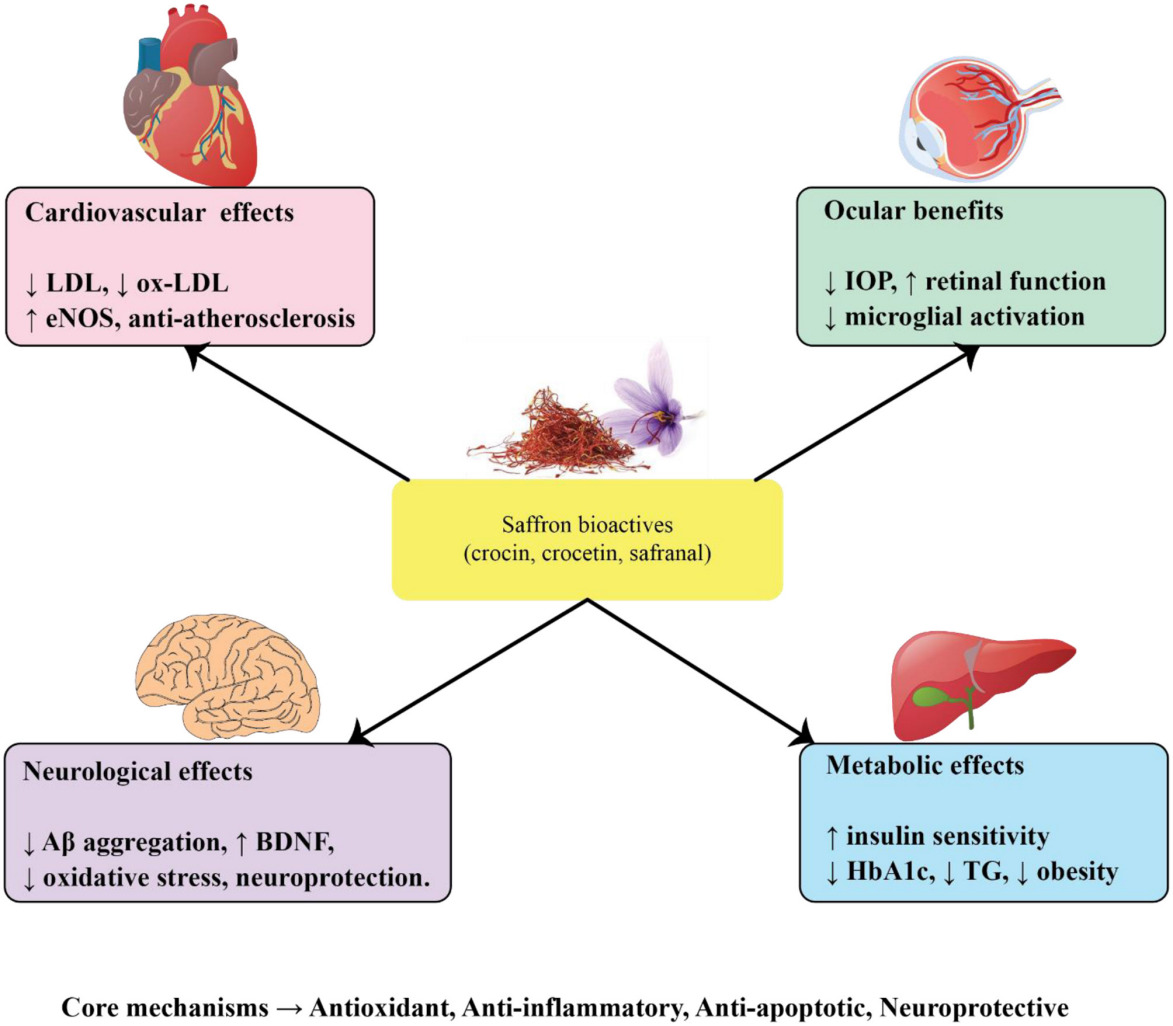
Mechanistic actions of saffron bioactives in age-related diseases.

The progressive rise in age-related disorders, including cardiovascular disease, diabetes, ocular pathologies, and neurodegenerative conditions such as Alzheimer's and Parkinson's disease, represents a major global health challenge. A central theme across these disorders is the interplay of oxidative stress, chronic inflammation, mitochondrial dysfunction, and apoptotic cell loss. Saffron (*Crocus sativus* L.) and its major bioactive constituents, crocin, crocetin, and safranal, have drawn increasing scientific attention due to their antioxidant, anti-inflammatory, neuroprotective, and metabolic regulatory properties. Evidence from preclinical studies and clinical trials indicates that saffron may exert multi-target benefits that extend across vascular, metabolic, visual, and neurological domains of aging (Figure 2). To provide an integrative overview, the following subsections summarize saffron's effects on cardiovascular diseases, metabolic disorders, age-related eye diseases, and neurodegenerative conditions. A consolidated summary of experimental and clinical findings across these domains is presented in Table 1.

**Table 1:** Summary of preclinical and clinical evidence on saffron and its bioactive compounds in age-related cardiometabolic, neurodegenerative, and ocular diseases.

**Model**	**Intervention**	**Main findings**	**Mechanisms**	**References**
**Cardiovascular diseases**				
Quails, endothelial cells, macrophages, SMCs	Crocin	↓ TC, TG, LDL-C; ↓ MDA; ↓ EC apoptosis; ↓ foam cells	Antioxidant, anti-apoptotic, inhibition of Ca^2^+-dependent SMC proliferation	(55)
Rabbits, BAECs	Crocetin	Restored endothelial relaxation; ↑ NO, ↑ eNOS, ↑ cGMP	eNOS upregulation, improved endothelial function	(56)
Rabbits	Crocetin	↓ VCAM-1 expression; ↓ NF-κB activation	Anti-inflammatory, endothelial protection	(57)
Rabbits	Crocetin	↓ ox-LDL, ↓ TBARS; ↑ antioxidant enzymes	Inhibition of LDL oxidation	(58)
Quails	Crocetin	↓ Lipids; ↓ oxidative stress; ↓ plaque formation	Hypolipidemic, antioxidant	(59)
CAD patients	Crocin, SAE	↑ SIRT1, AMPK; ↓ LOX1, NF-κB; ↓ ox-LDL, MCP-1	Anti-inflammatory, antioxidant gene modulation	(60)
Rats (ISO-MI)	Saffron extract	↓ Troponin I; preserved antioxidants; ↓ myocardial damage	Antioxidant, anti-necrotic	(61)
Rats (ISO-MI)	Crocin	Improved hemodynamics; ↑ SOD, CAT, GSH; ↓ MDA, necrosis	Antioxidant, redox balance	(62)
Rats (I/R)	Crocetin, crocetin ester	↓ Infarct size, ↓ cytokines, ↑ eNOS, ↑ Bcl-2	Anti-inflammatory, anti-apoptotic, Rho/ROCK/NF-κB inhibition	(63)
Rats (ISO-MI)	Safranal	↓ CK, LDH, MDA; ↑ SOD; improved Ca^2^+ handling	Antioxidant, Ca^2^+ regulation	(64)
WT and ApoE^−/−^ mice (I/R)	Saffron extract	↓ Infarct size; ↑ eNOS, Akt, ERK, Nrf2 signaling	Antioxidant, pro-survival signaling	(65)
Rat isolated hearts (I/R)	Crocin ± Vitamin E	Improved hemodynamics; ↓ infarct size	Antioxidant synergy	(66)
Rabbit hearts (DOX, I/R)	Saffron extract	Preserved cardiac proteins; ↓ ROS	Antioxidant, AKT/mTOR/4EBP1 activation	(67, 68)
H9c2 cells (DOX + I/R)	Saffron extract	↑ AKT/ERK signaling; ↓ apoptosis	Anti-apoptotic, mitochondrial protection	(69)
Rats, H9c2 cells (DOX)	Crocin	↓ CK, LDH; ↓ TNF-α, IL-6; ↓ TLR-2/NF-κB	Anti-inflammatory, mitochondrial stability	(70)
Rats (DOX)	Crocin	↓ Cardiac enzymes; ↓ apoptosis, inflammation	Antioxidant, anti-apoptotic	(71, 72).
Rats (Diazinon)	Crocin	↓ CK-MB, ↓ MDA; ↑ GSH; ↓ Bax/Bcl-2 ratio	Antioxidant, anti-apoptotic	(73, 74)
Mice (Zearalenone)	Crocin	↓ Cardiac markers, oxidative stress, apoptosis	Antioxidant, anti-apoptotic	(75)
**Diabetes and dyslipidemia**				
Metabolic syndrome patients (*n* = 44)	Saffron 100 mg/day, 12 wks	↓ TG, TC, LDL; ↑ HDL; ↓ FBG and hsCRP; modulation of cytokines (IL-6, VEGF, EGF)	Anti-inflammatory, lipid-lowering	(77)
Dyslipidemia patients (*n* = 40)	Saffron petal pills	↓ TG, TC, LDL; no change in FBS, ALT, AST, ALP	Hypolipidemic, renal protective	(76)
High-fat diet mice	Saffron extract, crocin, crocetin	↓ Cholesterol, TG, ROS; ↓ PCSK9 & sortilin; ↑ LDLR; ↓ SREBP-1c/2	PCSK9 inhibition, antioxidant	(82)
Hyperlipidemic rats	Saffron (25–100 mg/kg), Crocin (4.8–19 mg/kg)	↓ TG, TC, ALP, AST, ALT, MDA; ↑ SOD, CAT, FRAP	Antioxidant, hypolipidemic	(80)
Hyperlipidemic rats	Crocin 25–100 mg/kg, 10 d	↓ TG, TC, LDL, VLDL; ↑ fecal fat excretion	Pancreatic lipase inhibition	(81)
High-calorie diet rats	Saffron stigma/petal extract	↓ Weight, TG, insulin resistance; ↑ adiponectin; improved oxidative stress	Antioxidant, insulin-sensitizing	(86)
Type 2 diabetes patients (*n* = 64)	Saffron 30 mg/day, 3 mo	↓ FPG, HbA1c, TC, LDL, LDL/HDL ratio	Hypoglycemic, hypolipidemic	(83)
Type 2 diabetes patients (*n* = 54)	Saffron extract, 8 wks	↓ FBS; no significant effect on HbA1c, TG, LDL, HDL	Glycemic control	(84)
Diabetic maculopathy patients (*n* = 60)	Crocin 5 or 15 mg/day, 3 mo	Crocin 15 mg ↓ HbA1c, CMT, improved BCVA	Antioxidant, neuroprotective	(85)
Fructose-fed rats	Crocetin	Improved insulin sensitivity; ↑ adiponectin; ↓ TNF-α, leptin	Adipocytokine modulation	(48)
Metabolic syndrome patients (*n* = 60)	Crocin 30 mg/day, 8 wks	↓ Serum pro-oxidant/antioxidant balance; no change in FBG, lipids	Antioxidant	(87)
Type 2 diabetes patients (*n* = 80)	Saffron 100 mg/day, 12 wks	↓ Waist circumference, ↓ MDA; no effect on FBG, HbA1c, lipids	Antioxidant, anti-obesity	(88)
Metabolic syndrome patients (*n* = 44)	Crocin 30 mg/day, 8 wks	↑ CETP; no significant lipid/glucose effect	Lipid transfer modulation	(89)
Meta-analysis of RCTs (*n* = 6–14 trials)	Saffron, crocin, stigma extracts	↓ TC, TG; ↑ HDL; no effect on LDL, FBG	Pooled human evidence	(78, 79)
**Age-related eye diseases**				
Patients with mild/moderate AMD (*n* = 100)	20 mg/day saffron, 3 mo, crossover trial	Improved BCVA and mfERG responses; benefits also in patients using AREDS supplements	Antioxidant and neuroprotective activity enhancing retinal function	(90)
Early AMD patients (*n* = 29)	20 mg/day saffron, ~14 mo, open-label follow-up	Sustained improvement in flicker sensitivity and visual acuity over long term	Maintenance of macular function through antioxidant/neuroprotective pathways	(91)
AMD patients (*n* = 60, wet/dry)	30 mg/day saffron vs. placebo, 6 mo RCT	Significant ERG improvements at 3 mo; reduced OCT thickness in wet AMD	Functional retinal enhancement, reduced oxidative damage	(92)
AMD patients vs. lutein/zeaxanthin group	Saffron supplementation, ~29 mo follow-up	Stable visual function with saffron; deterioration in lutein/zeaxanthin group	Reduced microglial activation, modulation of protective genes	(93)
POAG patients (*n* = 34 eyes)	30 mg/day aqueous saffron, 1 mo	Significant IOP reduction compared with placebo; effect reversed after wash-out	Antioxidant action and ocular hypotensive effect	(97)
POAG patients (*n* = 40, 49 eyes)	15 mg/day crocin, 4 mo, RCT	Lowered IOP and improved cup-to-disc ratio; stabilized RNFL	Neuroprotection, antioxidant and anti-inflammatory effects	(98)
Ocular hypertension mice	Saffron extract standardized to crocin	Reduced microglial activation, preserved RGCs	Anti-inflammatory activity and neuroprotection	(99)
P23H rat (retinitis pigmentosa model)	Safranal supplementation	Preserved photoreceptor structure and vasculature; higher ERG responses	Anti-apoptotic and vascular-protective mechanisms	(94)
NMDA-induced retinal damage in mice	Crocetin 100 mg/kg orally	Reduced ganglion cell apoptosis, preserved ERG b-wave	Inhibition of caspase-3/7 pathway	(95)
Light-induced retinal degeneration in mice	Crocetin 100 mg/kg orally	Preserved photoreceptors and ERG function	Suppressed oxidative/ER stress; inhibition of caspase-3/9	(96)
Microglial cells (DR model)	Crocin treatment under HG/FFA conditions	Reduced oxidative stress and pro-inflammatory response	Activation of PI3K/Akt signaling; inhibition of inflammatory mediators	(100)
**Alzheimer's disease (AD)**				
Mild–moderate AD patients (*n* = 46)	Saffron 30 mg/day, 16 wks	Improved ADAS-cog and CDR scores vs. placebo; well-tolerated	Anti-amyloid, cognitive enhancement	(101)
Mild–moderate AD patients (*n* = 54)	Saffron 30 mg/day vs. donepezil 10 mg/day, 22 wks	Comparable efficacy to donepezil; fewer GI side effects	Cholinergic modulation, antioxidant	(102)
Mild–moderate AD patients (*n* = 60)	Saffron 30 mg/day + donepezil vs. donepezil alone, 12 wks	No extra cognitive benefit; ↓ IL-1β, ↓ MDA, ↑ TAC	Anti-inflammatory, antioxidant	(103)
Moderate–severe AD patients (*n* = 68)	Saffron 30 mg/day vs. memantine 20 mg/day, 12 mo	Similar efficacy in stabilizing cognition and function; safe	NMDA modulation, antioxidant	(104)
Systematic review of RCTs (*n* = 325)	Oral saffron (various doses)	Cognitive improvement vs. placebo; comparable to donepezil/memantine; well-tolerated	Multi-target effects (anti-amyloid, antioxidant)	(105)
PC12 cells (*in vitro*)	Crocin (10 μM)	↑ Glutathione synthesis; ↓ lipid peroxidation; ↓ caspase-3	Antioxidant, anti-apoptotic	(108)
Hippocampal Ht22 cells	Crocetin (1–5 μM) + Aβ1-42	↑ Cell viability; ↓ ROS; ↑ mitochondrial stability; ERK1/2 activation	Anti-oxidative, pro-survival signaling	(107)
PC12 cells (Aβ, H_2_O_2_ toxicity)	Safranal (2.5–5 μM)	↓ ROS, ↓ apoptosis; regulated MAPK and PI3K/Akt	Antioxidant, anti-apoptotic	(109)
Aβ42 fibril assay (*in vitro*)	Crocin	Prevented amyloid aggregation; disrupted preformed fibrils	Anti-amyloidogenic	(106)
PC12 and mouse models	Crocin vs. tricrocin vs. dicrocin	Crocin most potent in protecting neurons; ↑ GSH synthesis	Antioxidant, anti-apoptotic	(108)
**Parkinson's disease**				
*In vitro*: α-synuclein aggregation assays	Saffron extract, crocin-1, crocin-2, crocetin	Inhibited α-synuclein aggregation and promoted fibril dissociation; crocetin most potent	Direct anti-aggregation activity, fibril destabilization	(110)
Rat model (Rotenone-induced PD)	Crocin (30 mg/kg/day, i.p.)	Improved motor behavior; restored dopamine and TH; reduced α-synuclein	Activated PI3K/Akt/mTOR pathway; ↓GSK-3β, caspase-9; ↑miRNA-7, miRNA-221	(111)
PC12 cells (MPP+-induced injury)	Crocin	Preserved mitochondrial potential, ATP; reduced apoptosis; effective even with delayed treatment	Inhibited ER stress, calcium release; restored Wnt signaling; CHOP-dependent protection	(112)
Rat model (6-OHDA-induced PD)	Crocetin (25–75 μg/kg)	Improved locomotion; preserved dopamine and glutathione; ↓lipid peroxidation	Enhanced antioxidant enzymes (SOD, CAT); reduced TBARS	(113)
Mouse model (MPTP-induced PD)	Saffron pre-treatment (0.01% w/v in drinking water)	Prevented loss of dopaminergic neurons in SNc and retina	Preserved TH+ cells; antioxidant and anti-apoptotic effects	(114)
Drosophila PD model (mutant α-synuclein)	Saffron extract, crocetin	Improved climbing ability, extended lifespan, prevented retinal degeneration	Anti-aggregation, antioxidant protection	(18)
Drosophila (Rotenone-induced PD)	Saffron methanolic extract, crocin	Rescued locomotor function; restored dopamine; prolonged lifespan	Reduced oxidative stress, restored GSH; preserved mitochondrial function	(115)
Rat model (6-OHDA PD, spatial memory)	Saffron extract (5–10 μg/rat)	Restored spatial memory performance in Morris water maze	Antioxidant effect; reduced oxidative stress	(116)
Clinical trial (PD patients, *n* = 52)	Saffron (15 mg bid, 8 weeks, add-on therapy)	Improved depressive symptoms but no effect on motor function	Likely serotonergic/antioxidant pathways	(117)

### 3.1 Saffron and cardiovascular diseases

Cardiovascular diseases (CVDs) remain the leading cause of death worldwide, with atherosclerosis, myocardial infarction, and heart failure forming the major pathological outcomes. Increasing evidence indicates that saffron and its active constituents, particularly crocin, crocetin, and safranal, exert protective effects on cardiovascular health through their antioxidant, anti-inflammatory, and anti-apoptotic properties. Experimental studies in animals, cellular models, and recent clinical trials provide a solid basis for their role as complementary therapeutic agents in cardiovascular disorders. Crocin has been shown to reduce the development of atherosclerosis in quails fed with a hyperlipidemic diet. Its administration significantly lowered serum cholesterol, triglycerides, and LDL-C, decreased malondialdehyde (MDA), and prevented nitric oxide (NO) depletion. Mechanistically, crocin improved endothelial cell survival by preventing oxidized LDL (ox-LDL)–induced apoptosis, reduced cholesterol ester accumulation in macrophages, and inhibited smooth muscle cell proliferation by modulating intracellular Ca^2^^+^ signaling. Collectively, these actions limited foam cell formation and plaque development, key processes in atherosclerosis initiation and progression (55).

Crocetin, another carotenoid derivative, has also demonstrated vascular protection. In hypercholesterolemic rabbits, crocetin restored acetylcholine-mediated endothelium-dependent relaxation by upregulating endothelial nitric oxide synthase (eNOS) activity and increasing cGMP levels, while leaving endothelium-independent vasodilation intact (56). Additionally, crocetin supplementation suppressed vascular cell adhesion molecule-1 (VCAM-1) expression, a pivotal mediator of leukocyte recruitment during atherogenesis, through inhibition of NF-κB activation (57). These results highlight its dual role in improving endothelial function and attenuating vascular inflammation.

Another study confirmed that crocetin supplementation reduced oxidative modification of LDL, thereby lowering ox-LDL and thiobarbituric acid reactive substances (TBARS) while enhancing antioxidant enzyme activity in rabbits with diet-induced hyperlipidemia (58). Similarly, quail models confirmed crocetin's anti-atherogenic effects, showing decreased serum lipid levels, reduced oxidative stress, and suppressed plaque formation (59). Together, these findings indicate that saffron bioactives act at multiple stages of atherosclerosis by regulating lipid metabolism, protecting endothelial cells, and modulating vascular inflammation.

Beyond animal studies, clinical investigations have begun to explore saffron's cardiovascular benefits. A randomized controlled trial in coronary artery disease (CAD) patients demonstrated that 8 weeks of crocin supplementation (30 mg/day) significantly reduced serum ox-LDL and MCP-1 while upregulating SIRT1 and AMPK gene expression and downregulating LOX1 and NF-κB in peripheral blood mononuclear cells (60). These molecular changes indicate crocin's capacity to improve endothelial health and reduce inflammatory signaling in humans, supporting its translational potential for atherosclerosis management.

Saffron extracts and isolated constituents also display cardioprotective effects in myocardial injury models. In isoproterenol-induced myocardial infarction (MI) in rats, saffron administration decreased serum troponin I, preserved glutathione peroxidase activity, and reduced histopathological damage (61). Crocin supplementation in a similar model improved blood pressure, ventricular function, and antioxidant enzyme activity, while lowering MDA and myocardial necrosis, suggesting potent protective effects against oxidative stress–mediated cardiac damage (62). Crocetin has also been reported to ameliorate ischemia-reperfusion (I/R) injury in rats by reducing infarct size, attenuating inflammatory cytokines (TNF-α, IL-1β, and IL-6), and enhancing antioxidant defenses. Its cardioprotective effect was linked to inhibition of the Rho/ROCK/NF-κB pathway and upregulation of anti-apoptotic proteins such as Bcl-2 (63). Similarly, safranal showed protective effects in isoproterenol-induced MI by reducing oxidative stress markers, modulating intracellular Ca^2^^+^ homeostasis, and improving myocardial structure (64).

Several studies also highlight saffron's role in ischemia-reperfusion injury and drug-induced cardiotoxicity. In mouse models, saffron aqueous extract significantly reduced infarct size and oxidative damage via activation of Akt/eNOS/ERK and Nrf2 pathways (65). Crocin was shown to reduce infarct size and improve hemodynamic parameters in isolated rat hearts subjected to ischemia-reperfusion, with effects comparable to vitamin E, and an even stronger protection when combined (66). Cardioprotection has also been observed against anthracycline-induced cardiotoxicity. Saffron extracts reduced oxidative stress and preserved cardiac architecture in isolated rabbit hearts exposed to doxorubicin (67, 68). *In vitro*, saffron extract limited apoptosis and mitochondrial damage in cardiomyocytes subjected to combined ischemia-reperfusion and doxorubicin exposure, mainly by activating AKT/ERK pathways (69). Crocin specifically protected against doxorubicin-induced myocardial toxicity by downregulating TLR-2/NF-κB signaling and restoring mitochondrial potential in both *in vivo* and *in vitro* models (70). Other studies confirmed crocin's role in reducing doxorubicin-induced cardiac apoptosis and inflammatory infiltration by normalizing Bax/Bcl-2 ratios, caspase-3 activation, and cytokine imbalance (71, 72).

Saffron constituents have also been evaluated against toxic cardiovascular insults. Crocin mitigated diazinon-induced cardiotoxicity in rats by reducing oxidative stress, restoring glutathione levels, and preventing mitochondrial-mediated apoptosis (73, 74). Likewise, crocin protected cardiac tissue from zearalenone-induced oxidative stress and apoptosis in mice, reducing serum cardiac markers and improving antioxidant status (75).

Overall, saffron and its bioactive molecules protect against cardiovascular injury through multiple complementary mechanisms: lowering serum lipids, limiting LDL oxidation, improving endothelial function, modulating inflammatory signaling, and preserving antioxidant defenses. Preclinical models consistently demonstrate benefits in atherosclerosis, ischemia, and cardiotoxicity, while early clinical data confirm anti-inflammatory and endothelial-protective effects in CAD patients. These findings suggest that saffron could serve as a low-risk nutraceutical adjunct for cardiovascular prevention and therapy, though larger human trials are warranted.

### 3.2 Saffron and its constituents in dyslipidemia and diabetes

Saffron and its active carotenoids have been increasingly investigated as potential adjuncts for managing dyslipidemia and diabetes, two interrelated metabolic disorders that strongly predispose individuals to cardiovascular complications. Clinical and preclinical studies consistently show that saffron exerts hypolipidemic, antioxidant, and insulin-sensitizing effects, although outcomes vary depending on preparation, dosage, and study population.

Randomized clinical trials in humans highlight saffron's lipid-lowering potential. In patients with dyslipidemia, supplementation with saffron petal extract significantly reduced triglycerides, total cholesterol, and LDL cholesterol while simultaneously improving renal function indices, with no evidence of hepatic toxicity (76). Another clinical study in individuals with metabolic syndrome reported similar benefits, demonstrating decreased triglycerides and LDL cholesterol alongside increased HDL levels and reductions in inflammatory cytokines, including IL-6 and VEGF (77). Such findings suggest that saffron addresses not only lipid imbalance but also the underlying inflammatory milieu characteristic of metabolic disorders. Supporting these outcomes, a systematic review and meta-analysis of randomized controlled trials confirmed that saffron supplementation reduces total cholesterol and triglycerides while modestly elevating HDL, although effects on LDL remain inconsistent across studies (78, 79).

Animal research provides mechanistic explanations for these clinical effects. Oral administration of saffron and its main carotenoid, crocin, in hyperlipidemic rats decreased triglycerides, cholesterol, and liver enzyme markers of injury while enhancing antioxidant defenses, such as superoxide dismutase and catalase, thereby attenuating lipid peroxidation (80). Crocin specifically reduced serum triglycerides and cholesterol in a dose-dependent manner through selective inhibition of pancreatic lipase, limiting fat absorption and promoting fecal excretion of cholesterol (81). More recently, saffron, crocin, and crocetin were identified as natural inhibitors of proprotein convertase subtilisin/kexin type 9 (PCSK9), a central regulator of LDL receptor degradation. In high-fat diet–fed mice, these compounds reduced cholesterol and triglycerides, alleviated hepatic steatosis, and downregulated PCSK9 and sortilin expression while upregulating LDL receptors and suppressing transcription factors SREBP-1c and SREBP-2, collectively improving lipid clearance (82). This newly identified pathway positions saffron as a nutraceutical with effects comparable to pharmacological PCSK9 inhibitors.

In addition to lipid regulation, saffron influences glucose metabolism and insulin sensitivity. Clinical trials in patients with type 2 diabetes have shown promising outcomes. In a 3-month randomized trial, saffron supplementation significantly lowered fasting plasma glucose, HbA1c, total cholesterol, and LDL cholesterol compared with placebo, without adverse effects on hepatic or renal function (83). Another trial confirmed that saffron hydroalcoholic extract reduced fasting blood glucose over 8 weeks, though no significant improvements were detected in HbA1c or lipid levels (84). Crocin supplementation has demonstrated benefits in diabetic complications; in patients with diabetic maculopathy, crocin at 15 mg/day not only decreased HbA1c and fasting glucose but also improved ocular outcomes such as central macular thickness and visual acuity (85).

Preclinical findings provide biological support for these outcomes. In fructose-fed rats, crocetin improved insulin sensitivity by enhancing adiponectin expression and suppressing tumor necrosis factor-α and leptin, thereby restoring the balance of adipocytokines that regulate glucose homeostasis (48). In obese rats fed a high-calorie diet, saffron stigma and petal extracts reduced insulin resistance, lowered body weight, and improved oxidative stress and lipid profiles, with stigma showing a greater effect, likely due to its higher content of phenolic compounds (86). Moreover, crocin supplementation in metabolic syndrome patients reduced oxidative stress markers by significantly lowering serum pro-oxidant/antioxidant balance, even though no major changes in fasting glucose or lipid profile were observed (87).

Nevertheless, some human trials report modest or no significant improvements in certain markers. For example, saffron supplementation in type 2 diabetic patients reduced waist circumference and oxidative stress marker malondialdehyde but did not significantly influence fasting glucose, insulin sensitivity, or lipid profile compared with placebo (88). Similarly, crocin supplementation increased cholesteryl ester transfer protein levels but produced no meaningful improvements in lipids or fasting glucose in metabolic syndrome subjects (89). These mixed results highlight the variability in responses and underscore the importance of factors such as dosage, intervention duration, and bioavailability of saffron formulations.

Taken together, the evidence indicates that saffron and its bioactive compounds exert a broad spectrum of actions on metabolic health. By lowering triglycerides and cholesterol, inhibiting PCSK9-mediated LDL receptor degradation, reducing oxidative stress, and improving adipocytokine signaling, saffron offers a multifaceted approach to ameliorating dyslipidemia and enhancing insulin sensitivity. Although several clinical trials report promising results, heterogeneity across studies underscores the need for well-designed, longer-term investigations using standardized saffron preparations to clarify its role as an adjunct therapy in dyslipidemia and diabetes management.

### 3.3 Saffron and age-related eye diseases

Age-related eye disorders such as age-related macular degeneration (AMD), glaucoma, diabetic retinopathy, and inherited retinal degenerations remain among the most common causes of irreversible vision loss worldwide. A shared hallmark in the pathogenesis of these conditions is the contribution of oxidative stress, mitochondrial dysfunction, inflammation, and progressive neuronal cell death. Since saffron and its bioactive constituents (crocin, crocetin, and safranal) exhibit strong antioxidant, anti-inflammatory, and neuroprotective properties, they have been investigated as adjunctive therapies to slow disease progression and preserve visual function. Evidence from clinical and experimental studies provides a growing rationale for saffron supplementation in several age-related ocular diseases.

AMD is one of the leading causes of blindness in the elderly. Several randomized controlled trials have shown that saffron can improve retinal function and visual outcomes in patients with both dry and wet forms of AMD. In a crossover clinical trial with over 100 participants, oral saffron supplementation (20 mg/day) led to measurable improvements in best-corrected visual acuity (BCVA) and multifocal electroretinogram (mfERG) responses when compared with placebo. Importantly, these benefits were observed even in patients already using standard Age-Related Eye Disease Study (AREDS) supplements, suggesting that saffron provides additional advantages beyond conventional antioxidant therapy (90). Longer-term studies have confirmed that saffron's benefits are not transient. In a follow-up trial spanning more than a year, patients with early AMD receiving daily saffron supplementation maintained improved flicker sensitivity and visual acuity compared with baseline, and these changes remained stable throughout the observation period (91). Other randomized trials with 6-month treatment durations also demonstrated functional gains, particularly in electroretinographic outcomes and macular sensitivity. In patients with wet AMD, saffron use was associated with reduced retinal thickness and improved electrophysiological parameters, although some of these effects diminished at longer follow-up intervals (92).

Animal and cellular studies provide mechanistic evidence explaining saffron's protective effects. In light-induced retinal degeneration models, saffron treatment preserved photoreceptor integrity, reduced microglial activation, and maintained visual function. These effects were linked to the modulation of matrix metalloproteinase activity and stabilization of retinal architecture (93). In inherited retinal degeneration models, safranal delayed photoreceptor loss and preserved retinal vasculature, while electroretinogram recordings demonstrated preserved retinal responses in treated animals compared with untreated controls (94). Crocetin, another active component, has been shown to protect against excitotoxic damage in NMDA-induced retinal injury in mice. Oral administration reduced ganglion cell apoptosis, maintained b-wave amplitudes in ERG recordings, and inhibited caspase activation, supporting its anti-apoptotic and neuroprotective role (95). Similar effects were observed in models of oxidative and endoplasmic reticulum stress, where crocetin reduced cell death and preserved mitochondrial membrane potential, again highlighting its ability to counter stress-induced degeneration (96).

Glaucoma is a chronic optic neuropathy characterized by retinal ganglion cell (RGC) degeneration, often associated with elevated intraocular pressure (IOP). In a pilot trial involving patients with primary open-angle glaucoma (POAG), oral saffron supplementation (30 mg/day) for 1 month produced a significant reduction in IOP when used in combination with conventional topical medications. After discontinuation, IOP values gradually returned to baseline, indicating that the effect is treatment-dependent (97).

A more recent randomized, triple-blind trial with crocin supplementation in POAG patients confirmed these findings. Crocin (15 mg/day for 4 months) significantly lowered IOP and improved cup-to-disc ratio compared to placebo. While best-corrected visual acuity and retinal nerve fiber layer thickness were not significantly altered, disease progression was stabilized, suggesting a potential neuroprotective role (98). Complementary preclinical studies further support these clinical outcomes. In mouse models of ocular hypertension, saffron extracts reduced microglial activation, attenuated neuroinflammation, and prevented RGC loss, indicating that saffron's benefits extend beyond pressure control and include direct neuronal protection (99).

Diabetic retinopathy (DR) represents another major cause of visual impairment in aging populations. Crocin has been shown to counteract oxidative stress and inflammation in microglial cells exposed to high glucose and free fatty acid conditions, mimicking the diabetic environment. Through activation of the PI3K/Akt pathway, crocin suppressed pro-inflammatory mediators, reduced oxidative markers, and promoted neuronal survival (100). These findings indicate that saffron may provide protection against early inflammatory and neurodegenerative processes in DR, although more clinical validation is needed.

Taken together, both clinical and experimental data support saffron as a promising adjunct therapy for several age-related eye diseases. In AMD, consistent improvements in retinal function, visual acuity, and contrast sensitivity have been reported across trials. In glaucoma, saffron and crocin supplementation lower intraocular pressure and provide neuroprotection. Preclinical studies extend these observations to inherited retinal degenerations and diabetic retinopathy, showing broad neuroprotective and anti-inflammatory effects. Although results are encouraging, most clinical studies have relatively short follow-up periods and modest sample sizes. Long-term, large-scale trials are necessary to establish the durability of benefits and to optimize dosage regimens. Nevertheless, the current body of evidence positions saffron as a safe, low-risk, and biologically active nutraceutical that holds therapeutic potential in the management of degenerative eye diseases in aging populations.

### 3.4 Saffron and its potential in Alzheimer's disease

Alzheimer's disease (AD) is the most prevalent form of dementia, characterized by progressive memory loss, impaired cognition, and functional decline. Its neuropathology involves amyloid-β (Aβ) plaques, neurofibrillary tangles of hyperphosphorylated tau, oxidative stress, and chronic neuroinflammation, all of which contribute to synaptic dysfunction and neuronal death. Available pharmacological treatments, such as cholinesterase inhibitors (e.g., donepezil) and N-methyl-D-aspartate (NMDA) receptor antagonists (e.g., memantine), provide only symptomatic relief and do not halt disease progression. This has led researchers to explore natural compounds with multi-target neuroprotective properties. Among these, saffron and its constituents, crocin, crocetin, and safranal, have attracted considerable interest for their antioxidant, anti-amyloid, and anti-inflammatory activities.

Several randomized clinical trials have investigated saffron supplementation in patients with mild to moderate AD. One of the earliest studies demonstrated that 30 mg/day of saffron extract over 16 weeks significantly improved cognitive scores, measured by the Alzheimer's Disease Assessment Scale–Cognitive Subscale (ADAS-cog) and the Clinical Dementia Rating scale, compared with placebo. Importantly, saffron was well-tolerated, and adverse events did not differ from those in the placebo group, suggesting a favorable safety profile (101). A subsequent 22-week multicenter study compared saffron with donepezil, a standard therapy for AD. Results showed that saffron had comparable efficacy to donepezil in improving cognition, with similar adverse event rates, except for vomiting, which was more frequent in the donepezil group (102). This finding raised the possibility that saffron may offer equivalent therapeutic benefits with fewer gastrointestinal side effects.

More recent work has examined saffron in combination with existing medications. A randomized double-blind trial in patients receiving donepezil found that adding saffron did not significantly enhance cognitive scores beyond donepezil alone, as measured by the Mini-Mental State Examination (MMSE). However, saffron supplementation significantly reduced inflammatory markers such as IL-1β and oxidative stress marker malondialdehyde (MDA), while improving total antioxidant capacity (103). This suggests that saffron may provide adjunctive systemic benefits, even if the additive effect on cognition is modest in patients already on pharmacological therapy.

In patients with more advanced AD, saffron was also compared with memantine. A 12-month randomized trial found no significant differences between the two treatments in cognitive decline, as measured by the Severe Cognitive Impairment Rating Scale (SCIRS) and Functional Assessment Staging (FAST). Both groups experienced stabilization of symptoms, and adverse event rates were similar (104). These findings further strengthen the argument that saffron may be considered as a therapeutic alternative or complementary approach in AD management. A systematic review of randomized controlled trials, including more than 300 participants, concluded that saffron supplementation consistently improved cognitive outcomes compared with placebo and produced results comparable to donepezil and memantine. Importantly, saffron was well-tolerated, and no serious safety concerns were reported (105).

While clinical findings support saffron's symptomatic benefits, preclinical studies have provided valuable insight into the underlying mechanisms. Crocin, the most abundant water-soluble carotenoid in saffron, has been shown to inhibit the aggregation of Aβ42 fibrils and even disrupt preformed fibrils. *In vitro* assays using thioflavin T fluorescence and electron microscopy demonstrated that crocin reduced amyloid load and altered peptide conformation, favoring less toxic structures (106). Crocetin, another carotenoid from saffron, demonstrated protective effects against Aβ1-42-induced toxicity in hippocampal-derived cells. Treatment with crocetin improved mitochondrial membrane potential, reduced reactive oxygen species, and increased cell viability. Moreover, crocetin promoted extracellular signal-regulated kinase (ERK1/2) activation, a pathway involved in neuronal survival (107). Animal studies have also confirmed the neuroprotective potential of saffron constituents. In rodent models, crocin prevented cell death under oxidative and hypoxic stress by enhancing glutathione synthesis and inhibiting caspase-3 activation, thereby reducing apoptosis (108). Similarly, crocetin administration in mice exposed to neurotoxic stimuli prevented retinal and neuronal cell damage by modulating caspase pathways and reducing oxidative burden (96). Safranal, a volatile component of saffron, has also demonstrated protective activity against Aβ-induced toxicity. In PC12 cell models, safranal reduced ROS production, attenuated apoptosis, and modulated MAPK and PI3K/Akt signaling pathways, both of which play central roles in neuronal survival and synaptic plasticity (109).

Neuroinflammation and oxidative stress are recognized as central drivers of AD progression. Several studies have demonstrated that saffron reduces pro-inflammatory cytokines such as TNF-α and IL-6 while enhancing antioxidant defenses. Clinical data confirm these effects, with saffron supplementation lowering circulating markers of lipid peroxidation and boosting enzymatic antioxidants like superoxide dismutase (SOD) and glutathione peroxidase (GPx). This systemic improvement in redox balance may help slow neurodegeneration indirectly by reducing vascular and metabolic stressors that exacerbate AD pathology (103).

Saffron represents a safe and potentially effective complementary therapy for Alzheimer's disease. Clinical trials have shown that saffron supplementation improves cognitive outcomes in mild to moderate AD, with efficacy comparable to established drugs such as donepezil and memantine. Preclinical evidence highlights crocin, crocetin, and safranal as the key compounds mediating neuroprotection through anti-amyloid, antioxidant, and anti-inflammatory effects. Although larger and longer trials are necessary, current evidence positions saffron as a promising natural candidate for integrative management of Alzheimer's disease.

### 3.5 Saffron and Parkinson's disease

Parkinson's disease (PD) is a progressive neurodegenerative disorder marked primarily by the selective loss of dopaminergic neurons in the substantia nigra and the pathological accumulation of misfolded α-synuclein. These events lead to characteristic motor impairments, such as tremor, rigidity, and bradykinesia, as well as non-motor symptoms including depression and cognitive decline. Current pharmacological approaches, most notably levodopa and dopamine agonists, alleviate symptoms but do not halt the underlying neurodegeneration. This therapeutic gap has prompted growing interest in natural compounds with antioxidant, anti-inflammatory, and anti-apoptotic potential. Among these, saffron and its main bioactive components have been increasingly investigated for their neuroprotective properties in PD.

One of the hallmarks of PD pathology is the misfolding and aggregation of α-synuclein into toxic fibrils that form Lewy bodies. Inoue et al. (110) showed that saffron extracts and specific constituents, particularly crocin-1, crocin-2, and crocetin, were capable of both preventing α-synuclein aggregation and breaking down preformed fibrils. Using thioflavin T assays and electron microscopy, they observed that these molecules shortened and reduced fibrillar structures, with crocetin being the most potent. Such anti-aggregation effects suggest that saffron may intervene at an upstream stage of PD pathology by directly targeting misfolded proteins responsible for neuronal toxicity (110). Oxidative stress and apoptosis contribute heavily to dopaminergic cell death. Salama et al. (111) explored crocin's role in rotenone-induced PD in rats, a model that closely mimics the mitochondrial dysfunction observed in patients. Administration of crocin not only improved behavioral deficits but also activated the PI3K/Akt/mTOR signaling pathway, a critical regulator of neuronal survival. This modulation resulted in reduced activity of glycogen synthase kinase-3β (GSK-3β) and caspase-9, alongside decreased α-synuclein accumulation. Crocin also upregulated microRNA-7 and microRNA-221, which reinforced Akt/mTOR signaling. These findings indicate that crocin protects dopaminergic neurons through a multi-layered mechanism that integrates survival signaling, mitochondrial stabilization, and reduced apoptosis (111).

Mitochondrial dysfunction and endoplasmic reticulum (ER) stress are both implicated in PD progression. Experiments in MPP+-treated PC12 cells revealed that crocin preserved mitochondrial membrane potential, maintained ATP production, and reduced apoptotic cell death even when treatment was delayed after injury (112). These benefits were associated with suppression of ER stress markers, regulation of calcium release, and restoration of Wnt signaling pathways. Such findings underscore crocin's ability to interfere with multiple damaging cascades triggered by mitochondrial toxins, highlighting its therapeutic versatility. Crocetin, another major saffron carotenoid, has also shown efficacy in PD models. In a 6-hydroxydopamine (6-OHDA) rat model, crocetin treatment improved locomotor function, preserved dopamine levels in the striatum, and restored antioxidant enzyme activity in both the striatum and substantia nigra (113). Markers of lipid peroxidation were significantly reduced, and histopathological analysis confirmed preservation of nigral neurons. These effects support the notion that crocetin counteracts oxidative stress that drives dopaminergic cell death, thereby offering neuroprotection.

The neuroprotective effects of saffron have also been demonstrated in whole-animal models. In a mouse model of PD induced by MPTP, saffron pre-treatment prevented the typical loss of tyrosine hydroxylase-positive neurons in both the substantia nigra and the retina (114). Similarly, experiments in Drosophila overexpressing mutant forms of α-synuclein showed that saffron and crocetin preserved climbing ability, extended lifespan, and protected against retinal degeneration (18). Rao et al. (115) further confirmed these findings in a rotenone-induced fly model, showing that saffron extract and crocin reduced oxidative stress, restored glutathione, preserved dopamine levels, and delayed locomotor decline. While PD is primarily recognized as a motor disorder, patients often suffer from cognitive deficits and mood disturbances. In rat models, saffron extract improved spatial memory performance impaired by 6-OHDA injection (116). Clinically, adjunct saffron supplementation has been shown to significantly improve depressive symptoms in PD patients, though without significant benefit on motor outcomes (117). These results indicate that saffron may play a supportive role in addressing non-motor complications of PD, which substantially affect patients' quality of life. Evidence from *in vitro* experiments, animal models, and early-phase clinical trials strongly supports saffron's neuroprotective role in Parkinson's disease. Its bioactive constituents, crocin, crocetin, and safranal, target diverse pathological mechanisms, including α-synuclein aggregation, oxidative stress, mitochondrial dysfunction, ER stress, and inflammation. Additionally, saffron shows promise in alleviating depressive symptoms associated with PD. While larger clinical trials are required to determine its definitive therapeutic value, saffron appears to be a safe and promising adjunctive strategy for improving both neurological and psychological outcomes in PD.

## 4 Exercise and age-related health

**Figure 3:**
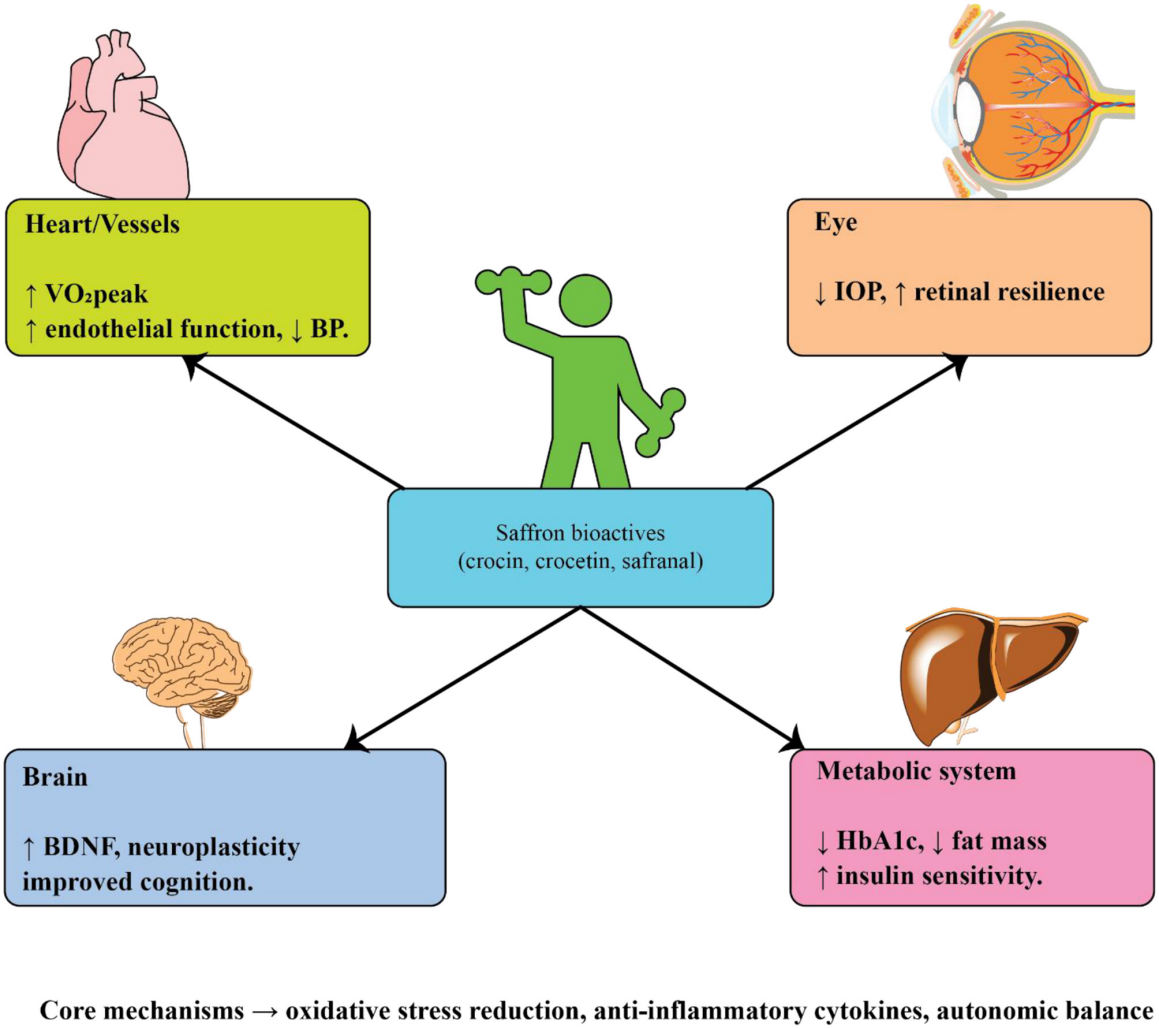
Systemic effects of exercise on age-related disorders.

Regular physical activity is one of the most effective lifestyle interventions for preventing and managing chronic age-related diseases. Beyond improving general fitness, structured exercise exerts wide-ranging benefits on cardiovascular function, metabolic regulation, cognitive performance, and psychological wellbeing. Evidence from epidemiological studies, randomized clinical trials, and experimental animal models consistently shows that exercise reduces morbidity and enhances quality of life in older populations. Mechanistically, these effects are mediated through improvements in autonomic regulation, mitochondrial function, neurotrophic support, vascular integrity, and inflammatory balance. Given the multidimensional role of exercise, its impact has been extensively studied in cardiovascular diseases, type 2 diabetes, age-related eye disorders, Alzheimer's disease, and Parkinson's disease (Figure 3). A comprehensive summary of key experimental and clinical findings across these conditions is provided in Table 2, which highlights the diversity of exercise modalities, patient populations, and mechanistic pathways investigated to date.

**Table 2:** Exercise modalities, outcomes, and mechanisms in age-related disease management.

**Model**	**Intervention**	**Main findings**	**Mechanisms**	**References**
**Cardiovascular diseases**				
Coronary artery disease patients	HIIT vs. MCT (8 weeks)	Both improved VO2peak, but HIIT gave greater gains; HRR significantly improved at 1–2 min	Enhanced parasympathetic reactivation, improved aerobic fitness	(118)
Women in cardiac rehab	Traditional CR vs. women-tailored CR (12 weeks)	Both improved HRR (1–6 min); predictors included baseline HRR, exercise capacity, anxiety	Parasympathetic recovery, reduced anxiety impact	(119)
Stable ischemic heart disease	Aerobic exercise vs. stress management vs. usual care (16 weeks)	Lower depression/distress, improved LVEF during stress, improved FMD	Better endothelial function, improved autonomic control	(120)
Heart failure with preserved EF (HFpEF)	Endurance training (16 weeks)	Increased VO2peak, improved QoL; no change in FMD or stiffness	Skeletal muscle/peripheral adaptations	(121)
Post-CABG patients	CR vs. home-based exercise vs. control (12 weeks)	CR improved HRR more than controls; home-based similar trend	Improved autonomic modulation	(122)
Post-CABG with VR	VR-enhanced aerobic training (3 months)	Higher VO2peak, METs, and anaerobic threshold vs. non-VR	Increased engagement, better exercise adherence	(139)
Post-AMI patients	3-month CR + continued training vs. standard advice	Sustained VO2peak and HRR improvements with continued training	Long-term autonomic balance	(124)
Coronary patients	Residential CR (cycle ergometer)	Improved HRR and BRS at rest	Enhanced baroreflex sensitivity	(125)
Extensive anterior AMI	Early exercise at ventilatory threshold	No LV remodeling improvement; possible aggravation	Incomplete ventricular healing	(126)
STEMI patients	CR started within 2 weeks (6 months)	Reduced ischemia, improved LV wall motion, higher VO2peak	Coronary vascular adaptation, collateralization	(227)
Heart failure post-PCI	Endurance vs. combined training (7 weeks)	Both ↑ functional capacity; ↓ NT-proBNP; hs-CRP reduced only in ET	Reduced wall stress, anti-inflammatory effects	(128)
Post-AMI patients (2 years)	Long-term CR with behavioral support vs. usual care	Maintained/improved VO2peak, lipid profile, ↓ events	Lifestyle adherence, metabolic control	(228)
Post-STEMI (6 months)	Early CR	Improved perfusion, LV wall thickness, VO2peak	Improved myocardial contractility and perfusion	(127)
Cardiac rehab with telemonitoring	FIT@Home (12 weeks) vs. center-based	Improved fitness, adherence, cost-effectiveness	Self-management, technology support	(129)
**Diabetes**				
Older adults with T2DM + knee OA	12 weeks dynamic resistance (elastic bands) vs. isometric training	Improved physical function and mobility (chair stand, TUG, WOMAC); no significant change in HbA1c	Enhanced muscle strength and balance	(134)
Adults with T2DM (*n* = 40)	4 months aerobic vs. resistance training	Both reduced HbA1c (~0.4%); aerobic improved VO2peak, resistance improved strength; reduced fat mass and increased insulin sensitivity	Improved cardiorespiratory fitness (aerobic) and muscle glucose uptake (resistance)	(130)
T2DM + coronary artery disease (*n* = 137)	1 year combined exercise training	No significant effect on HbA1c or VO2peak overall; benefits in subgroup without vascular complications	Exercise tolerance ↑; vascular status modifies response	(135)
T2DM patients (*n* = 39)	4 months strength vs. endurance training	Strength training ↓ HbA1c (8.3 → 7.1%), improved insulin resistance, lipids; endurance training less effective	Increased muscle glucose uptake, improved lipid metabolism	(131)
T2DM sedentary adults	4 months home-based resistance bands	No significant changes in HbA1c or function	Limited intensity insufficient for metabolic effect	(229)
T2DM (*n* = 75)	12 weeks aerobic training	↓ HbA1c, waist circumference, blood pressure, triglycerides; ↑ sRAGE, VO2peak	Improved insulin sensitivity, reduced inflammation, vascular protection	(139)
T2DM (*n* = 262)	9 months aerobic, resistance, or combined training	Only combined training ↓ HbA1c significantly; all groups reduced waist circumference	Synergistic effect of aerobic + resistance on glucose metabolism	(133)
T2DM adults	12 weeks aerobic, resistance, combined vs. control	Aerobic ↑ antioxidant enzymes (SOD, catalase), nitric oxide; ST improved HbA1c in poor baseline control	Reduced oxidative stress, improved insulin sensitivity	(230)
Middle-aged T2DM adults (*n* = 87)	12 weeks aerobic vs. meal replacement diet	Exercise improved diastolic function; diet improved glycemia and body composition	Cardiac remodeling & vascular stiffness reduced by exercise	(140)
Elderly T2DM (*n* = 300)	Progressive resistance training (bioDensity™) 6 months	No effect overall; subgroup with poor baseline HbA1c improved glycemia and lipids	High-intensity loading enhanced muscle metabolism	(145)
Overweight T2DM (*n* = 60)	6 months aerobic training	↓ HbA1c, insulin resistance, hsCRP, IL-18; ↑ IL-10	Anti-inflammatory and metabolic effects	(136)
T2DM (*n* = 54)	12 weeks aerobic training	Improved insulin sensitivity, ↑ apelin, ↑ ghrelin in women	Hormonal modulation of adipokines	(138)
T2DM (*n* = 100)	6 months aerobic, resistance, combined training	All improved HbA1c and insulin sensitivity; aerobic best for lipids, BP, CIMT	Anti-inflammatory, vascular protection	(137)
Older T2DM + hypertension + hyperlipidemia	3 months aerobic training	↓ arterial stiffness (radial, femoral PWV)	Vascular compliance improved independent of VO2max	(141)
T2DM (*n* = 96)	1 year HIIT+RT vs. MCT+RT	No HbA1c effect; MCT+RT improved body composition and CRF	Fat distribution and fitness improved with moderate training	(146)
T2DM (*n* = 32)	Paleolithic diet ± supervised exercise	Both ↓ HbA1c and insulin resistance; exercise preserved lean mass, ↑ VO2max	Diet-improved metabolic health, exercise-enhanced fitness	(147)
Elderly T2DM (*n* = 44)	12 weeks resistance training	↓ TNF-α and IL-1β; no significant HbA1c or endothelial function change	Partial anti-inflammatory effect	(231)
T2DM (*n* = 251)	22 weeks aerobic, resistance, combined	All ↓ HbA1c; combined training gave largest reduction (−0.5%)	Complementary effects on insulin sensitivity and muscle metabolism	(132)
Veterans with diabetic neuropathy (*n* = 45)	12 weeks aerobic, strength, or combined	No change in nerve conduction; modest sensory nerve improvement, some ↑ epidermal fiber density	Enhanced peripheral circulation, selective neuroprotection	(144)
Elderly T2DM (*n* = 39)	16 weeks aerobic training	↓ glucose excursions on OGTT, ↑ treadmill time, better attitudes toward DM	Improved glucose tolerance and psychosocial health	(143)
Newly diagnosed T2DM (*n* = 593)	Diet, or diet+activity vs. usual care	Diet and diet+activity ↓ HbA1c; activity added no extra benefit	Lifestyle change improves glycemia early in disease	(232)
T2DM (*n* = 173 from HART-D)	Aerobic, resistance, combined vs. control	Exercise improved physical QOL; combined training improved mental health and vitality	Physical and psychological wellbeing enhanced	(142)
**Age-related eye diseases**				
9,519 adults, 40–81 y (Aerobics Center Longitudinal Study)	Self-reported PA + treadmill fitness	Meeting PA guidelines (≥500 MET-min/week) or high fitness lowered glaucoma risk by ~40–50%	Reduced IOP, systemic vascular/metabolic benefits	(148)
24 glaucoma patients, ≥3 years follow-up	Habitual exercise (self-reported)	Exercisers had slower visual field defect progression	Independent of IOP, possibly neuroprotective	(150)
BALB/c mice, light-induced degeneration	Treadmill training (10 m/min, 1 h/day, 5 d/wk)	Exercised mice preserved double the photoreceptors and retinal function	↑ BDNF signaling; effect blocked by TrkB antagonist	(163)
UK Biobank participants (*n* > 80 k)	Accelerometer + self-reported PA	Higher PA linked with thicker macular GCIPL, no strong effect on glaucoma or IOP	Structural retinal benefits, modest systemic effects	(233)
Aged mice (12 mo) with IOP injury	Swimming 60 min/d, 5 d/wk	Exercise reversed age-related vulnerability of optic nerve	Reduced gliosis, normalized immune activation	(50)
Adult rats with optic nerve transection	Treadmill 30 min/d, 5 d/wk	More RGC survival at 5–7 days post-injury	↑ Retinal BDNF; blocked by TrkB antagonist	(154)
Naturally aged mice (22 mo)	12 wks treadmill (5–12 m/min)	Reversed retinal thinning and reduced oxidative stress markers	↓ Nitrotyrosine, ↓ CML, improved antioxidant status	(234)
Healthy humans (*n* = 45 intervention, *n* = 38 controls)	6-wk supervised aerobic + strength exercise	IOP reduced by ~2.2 mmHg in exercisers	Increased aqueous outflow, systemic hemodynamic effects	(152)
29 young healthy adults	20 min jogging	SC area, perimeter, and TM thickness increased; IOP reduced	Sympathetic activation, structural expansion of outflow pathways	(151)
141 glaucoma patients (mean age ~65)	Accelerometer-measured PA	More steps, MVPA, and nonsedentary time slowed VF loss	Neuroprotection; improved ocular perfusion	(149)
10,243 men ≥40 y (Korean survey)	Habitual PA	Vigorous exercise linked with lowest glaucoma prevalence	Non-IOP mechanisms suggested	(235)
29,854 male runners	Longitudinal PA and race pace	Faster 10 km pace and more km/week linked with lower glaucoma incidence	Fitness-related neuroprotection	(164)
U.S. NHANES cohort, diabetics (*n* = 282)	Sedentary behavior (accelerometer)	Each +60 min/d sedentary ↑ DR odds by 16%	Independent of PA; vascular dysfunction	(155)
40 DR patients	12-wk moderate aerobic exercise	↓ FBS and ↓ central macular thickness	Improved glycemic control, vascular health	(156)
Diabetic mice	Chronic exercise	Activated AMPK/miR-181b axis; improved endothelial function	Anti-inflammatory, ↑ NO signaling	(157)
Beaver Dam Eye Study (*n* = 3,874, 15-yr follow-up)	Self-reported PA	Active lifestyle reduced exudative AMD risk (OR 0.3)	Vascular, oxidative stress reduction	(158)
Melbourne Collaborative Cohort (*n* = 20,816)	Past vigorous exercise	Women exercising ≥3 × /week had lower intermediate AMD odds	Possibly sex-dependent vascular/oxidative effects	(159)
330 AMD patients + 121 controls	Lifestyle analysis	Higher PA associated with better VA and slower AMD severity	↑ Endogenous antioxidants; diet–exercise synergy	(160)
Mouse AMD models	Exercise *in vivo*	Reduced CNV and improved anti-VEGF efficacy	Inhibited AIM2 inflammasome via adiponectin/AMPK pathway	(161)
Rhodopsin mutant mice (adRP model)	Wheel running	Exercise preserved photoreceptors, RPE, and retinal thickness	↓ Apoptosis, ↓ inflammation, stabilized RPE	(162)
32,610 runners & 14,917 walkers (US cohorts)	Prospective PA	Both running and walking reduced cataract risk (up to 42%)	Energy expenditure–dependent, antioxidant pathways	(236)
52,660 Swedish adults	Long-term PA (various types)	High PA linked with 13% lower cataract risk; inactivity ↑ risk	Systemic metabolic and oxidative stress effects	(165)
17,777 Spanish adults	Cross-sectional PA survey	< 600 MET-min/week linked with higher cataract prevalence	Protective effect with ≥600 MET-min/week	(166)
**Parkinson's disease (PD)**				
26 patients with mild–moderate PD	3-month power yoga, 2 sessions/week	Reduced bradykinesia and rigidity; increased strength and mobility; improved quality of life	Neuromuscular strengthening, improved functional mobility	(186)
187 adults with PD	8 weeks mindfulness yoga vs. stretching & resistance training	Both improved motor scores; yoga reduced anxiety & depression, enhanced spiritual wellbeing and HRQoL	Mind–body integration, stress reduction	(187)
96 patients with PD	12-week tango, treadmill, or stretching	Treadmill improved forward walking; treadmill & stretching improved backward walking; tango showed limited effect	Task-specific gait adaptation	(188)
105 PD patients	10 sessions physiotherapy vs. treadmill	Improved gait speed, UPDRS-III, balance; similar effects between groups	Gait–cognition interaction, motor relearning	(189)
96 patients with mild–moderate PD	7 days accelerometer PA recording + neurocognitive tests	Moderate PA ≥150 min/week improved global cognition, executive and visuospatial function	Increased functional connectivity (hippocampus, frontal, parietal regions)	(190)
14 PD patients	1 session HIIT vs. moderate training vs. rest	HIIT improved memory, attention, sustained focus; MICT improved short-term memory only	Intensity-dependent neurocognitive activation	(191)
29 patients with PD	10 weeks HIIT vs. MICT cycling	Both ↑ VO_2_peak, ↓ motor symptoms, ↓ fatigue; HIIT showed larger gains in endurance	Improved cardiovascular fitness, reduced fatigability	(192)
20 PD patients	8 weeks aerobic interval training	Improved psychomotor control, executive function, bradykinesia	Neuroplasticity, executive control pathways	(193)
40 PD patients (Hoehn & Yahr 1–3)	9 weeks progressive resistance training	Reduced bradykinesia, improved walking, and sit-to-stand	Muscular strengthening, neuromuscular adaptation	(194)
35 elderly PD patients	24 weeks resistance training	Reduced anxiety, improved quality of life	Psychophysiological regulation	(195)
Single case (65 y/o male with PD)	6 weeks BFR treadmill walking	Improved gait, endurance, RLS symptoms	Enhanced perfusion, muscular adaptation under BFR	(196)
Single case (PD, recreationally active)	6 weeks BFR + resistance	Increased lower limb strength, improved RLS, QoL	Hypertrophy at low loads, reduced autonomic strain	(197)
38 PD patients	4 weeks LIRT-BFR vs. HIRT vs. control	BFR improved autonomic & endothelial function, BP, HRV	Vascular adaptation, autonomic regulation	(198)
Rat 6-OHDA model	14 days treadmill exercise	Improved motor tests; ↑ BDNF, TH; ↓ oxidative stress	Neurotrophic support, antioxidant defense	(199)
Rat 6-OHDA model	4 weeks treadmill training	Improved gait, restored mitochondrial dynamics	Enhanced fusion/fission, reduced oxidative stress	(200)
Mouse MPTP/P model	Treadmill exercise	↓α-syn accumulation; ↑ mitochondrial biogenesis & autophagy via SIRT1	SIRT1–PGC-1α signaling, autophagy	(201)
Mouse MPTP model	Endurance exercise	Restored motor deficits; ↑ neurogenesis, antioxidants, autophagy markers	Multi-pathway neuroprotection	(202)
Rats, PD model	8 weeks aerobic exercise	Improved motor behavior; ↑ TH, ↓α-syn; miR-3557 up, miR-324 down	CaMK–mTOR signaling, autophagy	(203)
Rats, PD model	Aerobic exercise	Reduced CAMKIIα carbonylation; restored apoptosis–autophagy balance	Protein homeostasis, autophagy regulation	(204)
Human RCT (*n* = 56)	6 months aerobic vs. stretching	↑ Connectivity in corticostriatal networks, reduced brain atrophy	Neural plasticity, improved cognitive control	(205)
Human RCT (*n* = 35)	36 aerobic exercise sessions	↑ Dopamine release in caudate; improved ventral striatum activity	Dopaminergic plasticity, reward circuitry	(206)
Human (*n* = 13 PD)	8 weeks body-weight supported treadmill	Improved MDS-UPDRS, QoL, 6-min walk	High-intensity gait retraining	(207)
Human RCT (*n* = 11 PD)	LSVT BIG vs. general exercise	Both ↓ UPDRS, depression, fatigue	Movement amplitude training, general mobility	(208)
Human RCT (*n* = 130 PD)	6 months home aerobic training vs. stretching	Aerobic group had greater ↓ motor UPDRS	Exergaming adherence, aerobic conditioning	(209)
**Alzheimer's disease (AD)**				
Older adults at risk (human)	24-week home-based physical activity	Modest cognitive improvement; slowed decline on ADAS-Cog	General neuroprotection via activity engagement	(167)
AD patients (human)	6-week non-aerobic movement program	Improved attention, visual memory; controls declined	Cognitive stimulation through movement	(168)
AD patients (human)	12-week home-based training (passive, motor-assisted, resistive)	Stabilized ADL and executive function; reduced caregiver burden	Combined motor and cognitive engagement	(169)
Mild AD (human)	16-week moderate–high intensity aerobic exercise	Reduced neuropsychiatric symptoms; cognitive benefit in adherent patients	Dose–response effect; improved mood regulation	(170)
Community-dwelling AD (human)	1-year home- or group-based exercise	Executive function improved with home-based training	Familiar environment may optimize adherence	(171)
Early AD (human)	6-month supervised aerobic training	Functional ability improved; fitness gains linked to hippocampal volume and memory	Cardiorespiratory fitness drives brain benefit	(172)
Mild–moderate AD (human)	6-month cycling vs. stretching	Both slowed expected decline; no between-group difference	General activity benefit, not modality-specific	(173)
Mild AD (human)	12-week aerobic + resistance vs. resistance alone	Both improved cognition and daily living; combined training strongest	Broader engagement of neural and muscular systems	(174)
Early AD (human)	6-month multicomponent sports training	Increased VO_2_max correlated with improved MoCA, executive function, hippocampal preservation	Fitness-related structural brain protection	(175)
APP/PS1 mice	12-week treadmill exercise	Reduced amyloid burden; improved synaptic markers; restored mitochondria	Enhanced mitophagy (PINK1/Parkin)	(178)
APP/PS1 mice	12-week treadmill exercise with SIRT1 modulation	Improved learning/memory; decreased plaques; rescued mitophagy	SIRT1–FOXO1/3 axis activation	(179)
APP/PS1 mice	8-week exercise ± chlorogenic acid	Combined EX+GCA superior for reducing plaques, oxidative stress, inflammation	SIRT1/PGC-1α pathway	(180)
APP/PSEN1 mice	5-month voluntary running	Preserved cognition; enhanced lysosomal clearance of Aβ	TFEB-mediated lysosomal biogenesis	(181)
3xTg-AD ovariectomized mice	3-month voluntary wheel running	Protected against memory loss, frailty, BPSD-like behaviors	BDNF-mediated neuroplasticity	(182)
Maternal swimming in rats	Prenatal and gestational exercise	Prevented offspring memory deficits, improved mitochondria	Mitochondrial biogenesis, synaptophysin upregulation	(183)
APP/PS1 mice	Treadmill exercise	Reduced Aβ via SIRT1 signaling; improved cognition	Shifted APP processing toward non-amyloidogenic pathway	(184)
Aged mice (perioperative model)	5-week resistance training	Reduced postoperative cognitive impairment and inflammation	PGC-1α/BDNF/Akt pathway; improved mitochondria	(237)
Rats (endurance & voluntary exercise)	Treadmill vs. free wheel	Improved mitochondrial respiration, decreased oxidative stress	Mitochondrial biogenesis, fusion, autophagy	(185)

### 4.1 Exercise and cardiovascular diseases

Cardiovascular diseases (CVD) remain the leading cause of morbidity and mortality worldwide, and their management increasingly incorporates lifestyle-based interventions alongside pharmacological care. Exercise training has been identified as a cornerstone in both primary and secondary prevention of coronary artery disease, myocardial infarction, and heart failure. The beneficial effects of exercise are mediated through improvements in autonomic function, myocardial perfusion, vascular function, metabolic capacity, and psychosocial wellbeing. Evidence from randomized clinical trials and rehabilitation studies strongly supports its role in enhancing prognosis and quality of life across a broad spectrum of cardiac patients. One of the most widely studied markers of autonomic balance and prognosis in cardiac patients is heart rate recovery (HRR) after exercise. HRR reflects parasympathetic reactivation following exertion and has been shown to predict long-term survival. Villelabeitia-Jaureguizar et al. (118) compared high-intensity interval training (HIIT) with moderate continuous training (MCT) in patients with stable coronary artery disease. Both programs significantly improved peak oxygen uptake (VO_2peak_), but HIIT induced larger gains in both VO_2peak_ and HRR at 1 and 2 min post-exercise, suggesting a superior effect on autonomic recovery and aerobic fitness. These findings highlight HIIT as a safe and more effective option than traditional continuous exercise in low-risk coronary patients (118). Similarly, Beckie et al.(119) examined women undergoing cardiac rehabilitation (CR) and found that both traditional and women-tailored CR programs improved HRR between 1 and 6 min after exercise cessation. Although no difference was observed between program types, both significantly enhanced autonomic recovery. Predictors of better HRR improvement included baseline HRR, higher exercise capacity measured in metabolic equivalents (METs), and reduced anxiety levels, whereas older age and insulin use were associated with poorer responses. These results emphasize the role of both physical capacity and psychosocial health in mediating the cardiovascular benefits of exercise (119).

Beyond HRR, exercise training also influences psychological wellbeing and vascular health in ischemic heart disease. A randomized trial by Blumenthal et al. (120) evaluated aerobic exercise and stress management in patients with stable ischemic heart disease. Compared with usual medical care, both exercise and stress management groups showed significant reductions in depression and general distress, smaller decreases in left ventricular ejection fraction during stress testing, and improved flow-mediated dilation. In addition, stress management enhanced baroreflex sensitivity and heart rate variability, further supporting the integrative benefits of behavioral interventions alongside exercise (120). In the setting of heart failure with preserved ejection fraction (HFpEF), exercise training has also demonstrated functional benefits. Kitzman et al. (121) randomized older patients with HFpEF to 16 weeks of endurance training or attention control. The exercise group showed significant improvements in VO_2peak_ and quality of life, although measures of endothelial function and arterial stiffness remained unchanged. These results indicate that gains in exercise capacity may be driven more by skeletal muscle adaptations and peripheral oxygen utilization than by central vascular changes in this population (121).

Post-surgical cardiac patients also benefit from structured exercise programs. Wu et al. (122) compared cardiac rehabilitation with home-based exercise after coronary artery bypass grafting (CABG). Both approaches improved HRR compared with baseline, but only the supervised CR group showed statistically greater improvements over the control group. This suggests that while home-based exercise is helpful, structured and supervised programs may yield superior autonomic recovery (122). Extending this concept, Chuang et al. (123) introduced virtual reality (VR) to enhance patient engagement in post-CABG exercise. Their study revealed that patients in the VR-enhanced group achieved higher VO_2peak_, METs, and anaerobic threshold values than those in conventional programs, supporting the role of novel technologies in optimizing rehabilitation outcomes (123).

The long-term impact of exercise training on autonomic recovery has also been explored. Giallauria et al. (124) studied patients after acute myocardial infarction (AMI) who completed a 3-month hospital-based exercise program. Those who continued structured home-based training maintained improvements in HRR and VO_2peak_ at 6 months, while those with only general advice experienced a decline. These findings underscore the need for ongoing, structured activity to preserve cardiovascular benefits beyond the initial rehabilitation period (124). Additional insights come from Legramante et al. (125), who found that residential cardiac rehabilitation improved HRR and baroreflex sensitivity in coronary artery patients. The parallel improvement in HRR and baroreflex measures further validates HRR as a simple, clinically meaningful marker of exercise-induced autonomic adaptation (125).

Exercise is not without caution. Kubo et al. (126) investigated patients with extensive anterior AMI and reduced ejection fraction, randomized to early supervised exercise at ventilatory threshold vs. standard care. While control patients showed reductions in left ventricular volumes, those in the exercise group exhibited no such improvement, and some measures suggested worsening remodeling. These results highlight the need for careful patient selection and timing, as exercise during early ventricular healing may aggravate adverse remodeling in severe infarcts (126).

Nonetheless, most studies support exercise as safe and beneficial when introduced appropriately. For example, Giallauria et al. (127) showed that beginning exercise rehabilitation within 2 weeks after STEMI reduced stress-induced myocardial hypoperfusion, improved wall motion and ejection fraction, and prevented unfavorable remodeling, leading to better functional recovery. Long-term programs combining educational and behavioral interventions, as evaluated by the same group, further demonstrated sustained improvements in VO_2peak_, lipid profile, and reduced clinical events over 2 years in post-AMI patients (127). Exercise also is proven to be effective in patients undergoing percutaneous coronary intervention (PCI). Abolahrari-Shirazi et al. (128) compared endurance training (ET) with combined endurance-resistance training (CT) in heart failure patients after PCI. Both exercise regimens significantly improved functional capacity and reduced NT-proBNP levels, while CRP decreased only in the endurance group. These findings indicate that both modalities are safe and effective, with subtle differences in inflammatory and biomarker responses (128). Recent strategies also seek to expand rehabilitation access through home-based and telemonitored approaches. The FIT@Home study compared 12 weeks of telemonitored home-based training with traditional center-based rehabilitation in low-to-moderate risk patients. Both approaches improved physical fitness and activity levels, but telemonitoring offered additional benefits in adherence, self-efficacy, and cost-effectiveness. This reflects a broader trend toward integrating technology to extend long-term rehabilitation support outside the hospital (129).

Taken together, these studies consistently demonstrate that exercise exerts beneficial effects on multiple domains of cardiovascular health. Improvements include enhanced autonomic recovery (HRR, baroreflex sensitivity), increased VO_2peak_ and functional capacity, better myocardial perfusion, reduced ischemia, improved left ventricular function, and favorable effects on psychological wellbeing. However, benefits depend on exercise modality, intensity, patient-risk profile, and timing of initiation. High-intensity interval training, when appropriately prescribed, appears particularly effective in improving aerobic fitness and autonomic recovery, whereas structured long-term programs are crucial for maintaining sustained cardiovascular protection. Thus, exercise training represents an essential non-pharmacological intervention in the prevention and management of cardiovascular diseases. Across diverse patient populations, from those recovering from myocardial infarction or bypass surgery to individuals with stable coronary artery disease or heart failure, structured physical activity improves autonomic balance, myocardial function, vascular health, and overall quality of life. While caution is warranted in high-risk or recently infarcted patients, the overall evidence strongly supports exercise as a safe, adaptable, and cost-effective component of cardiovascular care.

### 4.2 Exercise and type 2 diabetes

Type 2 diabetes mellitus (T2DM) is a chronic disease characterized by hyperglycemia, insulin resistance, and progressive β-cell dysfunction. Alongside pharmacological therapy, lifestyle interventions, particularly exercise, are considered essential for glycemic control and reduction of long-term complications. Different forms of physical activity, including aerobic, resistance, and combined modalities, have been studied for their effects on glycemic control, cardiovascular fitness, inflammation, and quality of life in patients with T2DM. The evidence shows that while results vary depending on exercise type, intensity, and patient comorbidities, structured training consistently improves metabolic health and functional outcomes. Both aerobic and resistance exercise offer benefits for patients with T2DM, but their effects on metabolic markers and functional capacity differ. A randomized trial compared aerobic and resistance training over 4 months and found that both modalities significantly reduced HbA1c by approximately 0.35–0.40%, with concurrent improvements in insulin sensitivity, lean body mass, and reductions in visceral and subcutaneous adiposity. Aerobic training more effectively enhanced cardiorespiratory fitness, while resistance training was superior for muscle strength. This suggests that each modality targets different components of physical health but provides similar metabolic improvements (130). A study by Cauza et al. (131) highlighted that resistance training might offer particular advantages. In their trial, patients who engaged in strength training demonstrated a significant reduction in HbA1c (from 8.3% to 7.1%), improved insulin sensitivity, and favorable changes in lipid profiles, including increased HDL and reduced triglycerides. By contrast, aerobic training did not produce significant changes in glycemic markers or lipids in that study, suggesting that resistance training may play a central role in metabolic management for patients with poor baseline control (131).

The integration of aerobic and resistance training may yield the most pronounced benefits. Sigal et al. (132) reported that combined training reduced HbA1c by 0.5% compared to sedentary controls, a greater reduction than achieved by aerobic or resistance training alone (132). Similarly, Church et al. (133) demonstrated that only the combined program significantly improved HbA1c, while single-modality training alone was insufficient. These results emphasize that combination training enhances glycemic control through complementary mechanisms, such as improving both cardiovascular efficiency and muscle glucose uptake (133).

The presence of comorbidities may influence exercise responses. Chen et al. (134) studied older patients with T2DM and knee osteoarthritis and compared dynamic vs. isometric resistance training. Both improved functional performance, but dynamic resistance training yielded superior outcomes in strength and mobility. However, neither intervention significantly altered HbA1c. This highlights that exercise improves quality of life and mobility even when direct glycemic effects are modest, particularly in populations with multiple chronic conditions (134). Similarly, Byrkjeland et al. (135) examined patients with T2DM and coronary artery disease and reported no overall significant improvement in HbA1c or VO_2peak_ after 1 year of combined training. Yet, patients without prior myocardial infarction or microvascular complications did show improvements, suggesting that the degree of vascular disease modifies exercise effectiveness. Importantly, even when glycemic benefits were limited, exercise improved ventilatory threshold and time to exhaustion, reflecting enhanced functional performance (135).

Chronic low-grade inflammation is closely linked with insulin resistance and cardiovascular risk in T2DM. Aerobic exercise appears particularly effective in reducing inflammatory markers. Kadoglou et al. (136) showed that 6 months of aerobic training reduced hsCRP and IL-18 while increasing IL-10, thereby shifting the balance toward an anti-inflammatory state. Another study by Kadoglou et al. (137) found that aerobic training reduced carotid intima-media thickness progression, which was independently associated with changes in inflammatory markers and improvements in VO_2peak_. These findings suggest that the vascular protective effects of exercise extend beyond glycemic control (137). Exercise also influences novel adipokines. Kadoglou et al. (138) demonstrated that aerobic exercise increased circulating apelin and improved insulin sensitivity, while ghrelin responses were modest and gender-dependent. These results highlight exercise-induced hormonal modulation as an additional mechanism supporting vascular and metabolic health (138).

Exercise interventions frequently improve body composition and cardiovascular function. Choi et al. (139) reported that aerobic exercise increased soluble receptor for advanced glycation end-products (sRAGE) and reduced body weight, waist circumference, and blood pressure, in parallel with decreased hsCRP. Improvements in sRAGE may provide vascular protection by neutralizing harmful AGE interactions (139). Gulsin et al. (140) further showed that supervised aerobic training improved diastolic function in middle-aged adults with T2DM, even without substantial weight loss, underscoring the independent cardiovascular benefits of exercise (140). A trial by Madden et al. (141) demonstrated that 3 months of aerobic training reduced arterial stiffness in older adults with T2DM and comorbid hypertension and hyperlipidemia. Notably, these benefits occurred without significant improvement in VO_2max_, suggesting that vascular adaptations may occur independently of cardiorespiratory capacity (141).

Exercise also enhances daily functioning and overall quality of life (QOL). Myers et al. (142), in the HART-D study, showed that aerobic, resistance, and combined training improved physical health scores compared to controls, with combined training producing additional benefits in vitality and mental health domains. Improvements in physical function are especially important in older diabetic adults, who face an increased risk of disability (142). Tessier et al. (143) also demonstrated that aerobic exercise reduced glucose excursions during oral glucose tolerance testing and improved attitudes toward diabetes in older participants, suggesting psychosocial as well as metabolic benefits (143). Exercise may help preserve nerve function in diabetic neuropathy. Stubbs et al. (144) studied veterans with length-dependent polyneuropathy and found that while nerve conduction parameters were largely unchanged, exercise modestly improved sensory nerve function and, in some cases, epidermal nerve fiber density. This suggests potential neuroprotective effects that require confirmation in larger studies (144).

Novel training protocols are being explored to enhance outcomes. Hangping et al. (145) tested a high-intensity progressive resistance training method (bioDensity™) in older Chinese patients. While overall effects on HbA1c were not significant, patients with poor baseline control (HbA1c >7.5%) showed meaningful improvements, as well as favorable lipid changes (145). Similarly, Magalhães et al. (146) compared high-intensity interval training (HIIT) plus resistance training vs. moderate-intensity continuous training plus resistance training. Neither improved HbA1c, but moderate continuous training improved body composition and fitness, reinforcing that not all exercise intensities translate into glycemic benefits (146).

Otten et al. (147) combined a Paleolithic diet with or without supervised exercise and found that both groups improved insulin sensitivity and HbA1c, but exercise preserved lean mass and increased cardiovascular fitness, indicating an additive role of physical activity in structured lifestyle programs. Collectively, evidence supports the role of exercise as a cornerstone of type 2 diabetes management. Aerobic and resistance training independently improve glycemic control, insulin sensitivity, and body composition, while combined training offers the most consistent improvements in HbA1c. Beyond metabolic effects, exercise reduces inflammation, improves vascular health, enhances functional performance, and contributes to a better quality of life. While outcomes vary depending on comorbidities and baseline metabolic health, structured and sustained physical activity remains one of the most effective non-pharmacological strategies for improving overall outcomes in individuals with type 2 diabetes.

### 4.3 Exercise and age-related eye diseases

Age-related eye disorders such as glaucoma, diabetic retinopathy (DR), age-related macular degeneration (AMD), and cataracts are among the leading causes of visual impairment and blindness in older adults. Increasing evidence highlights physical activity as a low-cost, accessible intervention with the potential to preserve visual health by modulating intraocular pressure (IOP), retinal metabolism, oxidative stress, and neurotrophic signaling. Findings from epidemiological studies, clinical trials, and experimental animal models collectively suggest that exercise exerts protective effects across multiple ocular pathologies, though the mechanisms and magnitude of benefits vary between diseases.

Glaucoma is strongly linked to age and characterized by progressive optic neuropathy and visual field (VF) loss. Physical activity appears to influence both disease onset and progression. In a large prospective cohort (n ≈ 9,500 adults, mean age 50), individuals meeting weekly activity recommendations (>500 MET-min/week) had nearly half the risk of developing glaucoma compared with inactive peers. High cardiorespiratory fitness, measured by treadmill testing, further reduced risk, and the combination of both behaviors conferred the lowest hazard ratio (148). Other longitudinal studies reinforce these associations. Among older adults with glaucoma, accelerometer-measured steps and time in moderate-to-vigorous activity correlated with slower VF deterioration. Specifically, an additional 5,000 steps per day or >2.5 h of non-sedentary activity reduced VF loss by approximately 10% (149). Retrospective work further showed that patients who reported regular exercise experienced slower progression of glaucomatous visual field defects, even when mean IOP values were similar between exercisers and non-exercisers, suggesting benefits beyond pressure reduction (150).

Mechanistic investigations help explain these findings. Jogging and other aerobic activities acutely reduce IOP, likely through increased aqueous outflow mediated by sympathetic stimulation and expansion of the trabecular meshwork and Schlemm's canal (151). Controlled interventions in healthy volunteers show that 6 weeks of supervised aerobic and strength training lowered mean IOP by more than 2 mmHg, while no changes were seen in controls (152). Animal studies provide additional mechanistic depth: treadmill and swimming exercise in aged rodents enhanced resilience of the optic nerve to pressure injury, reduced retinal gliosis, and normalized macrophage activation (153, 154). Collectively, these data indicate that exercise protects retinal ganglion cells (RGCs) through both pressure-dependent and neurotrophic mechanisms, the latter largely mediated by exercise-induced increases in BDNF.

DR is a microvascular complication of diabetes that accelerates with age and poor metabolic control. Physical activity may play dual roles in improving systemic glucose regulation and protecting retinal tissue directly. Cross-sectional U.S. data revealed that sedentary behavior independently predicted higher odds of DR in adults with diabetes, even after adjusting for total activity and glycemic parameters (155). This suggests that prolonged inactivity may contribute to microvascular damage irrespective of exercise levels. Clinical trials confirm the benefits of structured aerobic exercise. In a 12-week intervention with moderate-intensity training, patients with non-proliferative DR experienced significant reductions in fasting blood glucose and central macular thickness, both markers of disease progression (156). Exercise-induced improvements in systemic metabolism, such as enhanced insulin sensitivity and reduced vascular inflammation, are thought to underlie these retinal outcomes. Preclinical studies support these mechanisms: in diabetic mice, endurance training activated the AMPK/miR-181b signaling axis, which alleviated endothelial dysfunction and reduced inflammatory markers relevant to DR progression (157). Together, these findings highlight exercise as a modifiable factor capable of reducing both systemic and ocular risks in diabetic patients.

AMD, particularly its neovascular form, is a major cause of irreversible blindness in older populations. Early population-based data from the Beaver Dam Eye Study showed that individuals reporting regular physical activity at baseline were less likely to develop exudative AMD 15 years later (158). Similarly, the Melbourne Collaborative Cohort Study linked frequent vigorous activity, particularly in women, with reduced odds of intermediate AMD (159). Lifestyle analyses in AMD patients further demonstrate that higher physical activity scores are associated with better visual acuity and slower disease progression, likely due to enhanced antioxidant defenses and reduced oxidative stress (160). Mechanistic work in mouse models indicates that exercise reduces choroidal neovascularization by inhibiting AIM2 inflammasome activation in myeloid cells. Serum from exercised animals transferred similar benefits to sedentary counterparts, suggesting that systemic mediators, including adiponectin, may play a role (161). These findings collectively point to exercise as a protective factor in AMD, possibly delaying onset and enhancing responses to anti-VEGF therapies.

Beyond AMD and DR, exercise has shown neuroprotective effects in inherited and light-induced retinal degeneration models. Wheel running in mice carrying rhodopsin mutations (I307N) prevented photoreceptor apoptosis, preserved retinal thickness, and reduced inflammatory responses (162). Earlier studies demonstrated similar protection in light-induced degeneration, with exercised animals retaining twice as many photoreceptors compared to sedentary controls, an effect abolished by blocking BDNF signaling (163). These data strongly suggest that systemic exercise elevates neurotrophic support within the retina, contributing to resilience against degenerative stress.

Cataracts are the leading cause of blindness worldwide and are closely tied to aging. Epidemiological studies show that habitual walking and running are associated with reduced cataract risk. In large cohorts of U.S. runners and walkers, cataract incidence declined linearly with increasing exercise energy expenditure, with up to a 42% lower risk among the most active individuals (164). Similarly, Swedish population-based data demonstrated that long-term high physical activity, particularly occupational activity and regular walking or cycling, was linked with a 13–24% decreased risk of cataract (165). Spanish survey data confirmed that meeting WHO activity guidelines (>600 MET-min/week) was associated with lower cataract prevalence, especially in older age groups (166). Although mechanisms are not fully defined, exercise may prevent cataracts by improving systemic antioxidant capacity, reducing oxidative stress in the lens, and lowering chronic inflammation, factors known to contribute to lens opacification.

Across ocular conditions, several common mechanisms appear to underlie exercise benefits. These include acute and chronic reductions in IOP, improved ocular blood flow, upregulation of BDNF and other neurotrophic factors, attenuation of oxidative stress, and suppression of inflammatory signaling. Exercise also indirectly benefits the eye by improving systemic metabolic control, vascular health, and reducing sedentary behavior, all of which influence ocular aging. Evidence from both human and animal studies strongly supports exercise as a protective factor against multiple age-related eye diseases. Regular physical activity reduces glaucoma risk and slows its progression, attenuates DR severity through improved glycemic and vascular function, decreases AMD incidence and progression by modulating oxidative and inflammatory pathways, and lowers cataract risk through systemic antioxidant mechanisms. Experimental models consistently show neuroprotective and anti-inflammatory effects mediated by factors such as BDNF and adiponectin. Although not a substitute for medical treatment, exercise represents a powerful and accessible tool to preserve visual function and delay the onset of vision-threatening diseases in aging populations.

### 4.4 Exercise and Alzheimer's disease

Alzheimer's disease (AD) is the most common cause of dementia, characterized by progressive decline in memory, cognition, and functional abilities. While pharmacological approaches provide modest symptomatic relief, they do not halt disease progression. In recent decades, physical exercise has emerged as a promising non-pharmacological intervention to slow cognitive decline and improve quality of life. Findings from randomized clinical trials, pilot studies, and animal experiments offer growing evidence that different forms of physical activity, ranging from aerobic training to multimodal and resistance-based programs, can provide both functional and neurobiological benefits in AD. Early clinical evidence came from the randomized controlled trial by Lautenschlager et al. (167), who investigated a 6-month home-based physical activity program in older adults with subjective memory complaints. Compared with controls, participants in the intervention group experienced modest improvements on the Alzheimer's disease Assessment Scale–Cognitive Subscale (ADAS-Cog) that persisted at 18 months. Although the effect size was small, it suggested that regular exercise can slow cognitive deterioration even before dementia is diagnosed (167). Other trials in patients with diagnosed AD have supported these findings. Yágüez et al. (168) tested a 6-week non-aerobic movement program in individuals with Alzheimer-type dementia and found improvements in attention and visual memory. Importantly, patients in the control group declined over the same period, while those exercising maintained or enhanced cognitive performance, underlining the protective effect of even short interventions (168).

A pilot randomized controlled trial by Holthoff et al. (169) extended these results by showing that home-based physical activity not only improved executive function and daily living skills but also stabilized caregiver burden. Patients engaged in structured leg and movement training demonstrated better outcomes than those receiving standard care, indicating potential transfer benefits from motor activity to daily functioning and caregiver quality of life (169). The role of exercise intensity has also been examined. In the largest trial to date, Hoffmann et al. (170) randomized 200 patients with mild AD to supervised moderate-to-high intensity aerobic exercise or usual care. While intention-to-treat analysis did not show global cognitive benefits, patients who adhered closely to the exercise program improved on the Symbol Digit Modalities Test, suggesting a dose–response effect. Notably, exercise also reduced neuropsychiatric symptoms such as agitation and mood disturbances, which are highly burdensome in AD care (170). Long-term interventions highlight that sustained engagement matters. The Finnish Alzheimer's Disease Exercise Trial by Öhman et al. (171) evaluated 1 year of either home-based or group-based training. Although global cognition did not significantly differ across groups, home-based exercise improved executive function compared with controls, suggesting that familiar environments and individualized routines may maximize adherence and benefit (171).

Aerobic-focused studies offer additional insights. Morris and Vidoni (172) showed that 6 months of supervised aerobic training improved functional ability, and changes in cardiorespiratory fitness correlated with memory performance and hippocampal volume preservation. This link between fitness gains and brain outcomes suggests that physiological adaptation, rather than exercise alone, drives cognitive resilience (172). Similarly, Yu et al. (173) reported that 6 months of cycling attenuated expected declines in ADAS-Cog scores, although differences with stretching controls were not statistically significant. Nevertheless, both groups performed better than the natural progression of AD, pointing to a general benefit of structured activity (173). More recent work has emphasized multimodal programs. Papatsimpas et al. (174) demonstrated that combining aerobic and resistance training for 12 weeks significantly enhanced global cognition and daily function compared with controls, while resistance training alone provided more modest improvements. This highlights the value of diverse training modes targeting both strength and endurance (174). Likewise, David et al. (175) showed that increases in cardiorespiratory fitness during a 6-month multicomponent sports intervention correlated with better executive function, higher MoCA scores, and preservation of hippocampal volume, reinforcing the neuroprotective role of physical fitness (175). Other controlled studies have found less consistent cognitive outcomes but still report functional or psychosocial benefits. Toots et al. (176) observed no significant cognitive improvement from high-intensity exercise in nursing home residents with dementia, while Henskens et al. (177) noted gains in mobility, endurance, and mood, particularly when training incorporated daily life activities. Together, these findings suggest that population characteristics, stage of disease, and type of training may influence outcomes (176, 177).

Preclinical models provide strong support for the neurobiological mechanisms underlying exercise benefits in AD. Transgenic mouse and rat models consistently demonstrate that aerobic or resistance training reduces amyloid-β burden, improves synaptic function, and enhances mitochondrial quality. For example, treadmill running in APP/PS1 mice reduced amyloid plaque deposition, restored mitochondrial structure, and increased synaptic proteins, such as synaptophysin and GAP43 (178). Follow-up work showed that these effects were mediated by activation of the PINK1/Parkin mitophagy pathway via the SIRT1–FOXO axis, highlighting a link between exercise, mitochondrial clearance, and neuronal survival (179). Complementary evidence comes from combined lifestyle interventions. Shi et al. (180) demonstrated that exercise plus chlorogenic acid supplementation in AD mice synergistically reduced oxidative stress, neuroinflammation, and amyloid accumulation through activation of the SIRT1/PGC-1α pathway, producing greater improvements than either intervention alone (180). Similarly, Wang et al. (181) reported that long-term voluntary running enhanced lysosomal function and amyloid clearance via TFEB-mediated gene transcription, leading to significant cognitive improvements in APP/PSEN1 mice.

Sex- and hormone-specific models have also been examined. In ovariectomized 3xTg-AD mice, exercise protected against memory loss and behavioral deficits by upregulating BDNF signaling, suggesting particular benefits for postmenopausal women at risk for AD (182). Maternal exercise during pregnancy has even been shown to protect offspring from amyloid-induced neurotoxicity in adulthood, through long-lasting enhancements in mitochondrial function and synaptic plasticity (183). Mechanistically, across studies, exercise consistently upregulates neurotrophic factors (BDNF, PGC-1α), enhances mitochondrial biogenesis and turnover, decreases oxidative stress, and promotes non-amyloidogenic APP processing through SIRT1-mediated signaling (184, 185). These converging pathways demonstrate how physical activity directly counteracts hallmark pathologies of AD. Overall, the evidence from human and animal studies demonstrates that physical exercise offers meaningful benefits for individuals with Alzheimer's disease. While not all trials show consistent effects on global cognition, improvements are frequently seen in executive function, daily living skills, neuropsychiatric symptoms, and caregiver burden. Mechanistic studies strongly support that exercise promotes brain resilience by reducing amyloid accumulation, enhancing mitochondrial health, and boosting neurotrophic signaling. Collectively, these findings underscore that structured physical activity, whether aerobic, resistance-based, or multimodal, represents a practical and powerful adjunct therapy to slow functional decline and improve quality of life in people living with AD.

### 4.5 Exercise and Parkinson's disease

Parkinson's disease (PD) is a progressive neurodegenerative disorder characterized by tremor, rigidity, bradykinesia, postural instability, and a broad range of non-motor symptoms, including depression, fatigue, and cognitive impairment. While pharmacological treatments remain the mainstay of care, exercise has emerged as a valuable adjunct that can alleviate symptoms, enhance function, and possibly influence disease progression. Evidence from clinical and preclinical studies highlights how structured exercise, from yoga and resistance training to treadmill walking and high-intensity aerobic interventions, improves motor outcomes, quality of life, and even neurobiological markers of PD.

Mind–body exercises, such as yoga, provide both motor and psychological benefits. A controlled trial showed that a 3-month power yoga program significantly reduced bradykinesia and rigidity while improving muscle strength, leg power, and daily mobility scores in older adults with PD (186). Beyond motor function, mindfulness-based yoga yielded additional benefits. In a large randomized trial, yoga was more effective than stretching and resistance training in reducing anxiety and depression, while also improving spiritual wellbeing and health-related quality of life (187). These results suggest yoga is a holistic intervention capable of addressing both physical and emotional aspects of PD. Comparisons among different exercise modalities have produced valuable insights. In one head-to-head trial, treadmill training led to sustained improvements in forward gait, while backward walking improved with treadmill and stretching but not with tango dance, contrary to expectations (188). Other investigations confirmed that treadmill training and individualized physiotherapy both enhance gait speed and stability during dual-task conditions, which are critical for fall prevention (189). Collectively, these findings reinforce the role of walking-based interventions in improving functional mobility in PD patients.

Cognitive impairment is a common complication of PD. Observational work found that patients who engaged in moderate-intensity physical activity, meeting at least 150 min per week as recommended by WHO, performed better in executive function, visuospatial ability, and memory. These benefits correlated with stronger functional connectivity between the hippocampus, brainstem, and frontal–parietal regions (190). Short-term studies also suggest intensity matters: acute moderate training enhanced short-term recall, while high-intensity interval training (HIIT) produced broader effects on attention and sustained focus (191). Thus, aerobic activity can mitigate cognitive decline in PD, likely through neuroplastic adaptations in corticostriatal and cortical networks. The optimal intensity of aerobic training remains debated. In a recent randomized trial, both HIIT and moderate continuous training improved cardiorespiratory fitness and motor symptoms, but HIIT achieved a larger, clinically meaningful increase in peak oxygen uptake and reduced knee extensor fatigability (192). Another study found that aerobic interval training improved psychomotor function, executive ability, and manual dexterity after 8 weeks (193). These data indicate that both modes are beneficial, with HIIT potentially offering additional advantages in endurance and strength.

Resistance training has shown strong effects on bradykinesia and muscle weakness, two disabling features of PD. Nine weeks of progressive resistance training improved motor scores, walking performance, and sit-to-stand tests (194). A 24-week program further reduced anxiety symptoms and enhanced quality of life in elderly PD patients (195). Novel approaches such as blood flow restriction (BFR) training are also promising. Case-based and controlled studies reported that low-load resistance exercise with BFR improved lower limb strength, walking endurance, and autonomic function, while reducing symptoms of restless leg syndrome (196–198). Compared to high-intensity resistance training, low-intensity BFR was equally or more effective in improving vascular and autonomic markers, offering a safer option for frailer patients.

Preclinical studies shed light on the mechanisms through which exercise exerts neuroprotection. In rodent PD models, treadmill and endurance training improved motor performance, preserved dopaminergic neurons, reduced α-synuclein accumulation, and enhanced mitochondrial function (199–202). These effects were associated with antioxidant upregulation, autophagy activation, and mitochondrial quality control, mediated in part by SIRT1-PGC-1α signaling and lysosomal pathways. Regular aerobic activity also altered microRNA expression (upregulating miR-3557, downregulating miR-324), which modulated CaMK and mTOR signaling cascades to promote neuroprotection (203, 204). Human neuroimaging adds convergent evidence. Aerobic exercise increased functional connectivity between the putamen and motor cortex, reduced brain atrophy, and improved executive control tasks (205). PET imaging demonstrated enhanced dopamine release in the caudate nucleus and increased striatal responsiveness during reward tasks after aerobic training (206). These findings suggest that exercise not only improves symptoms but also promotes dopaminergic neurotransmission and corticostriatal plasticity.

Innovative protocols further expand exercise's role in PD. Body-weight–supported treadmill training allowed participants to achieve high-intensity walking safely and resulted in improved motor scores, quality of life, and walking distance (207). Similarly, LSVT BIG therapy, designed to encourage exaggerated movement patterns, produced improvements in motor and non-motor symptoms comparable to general exercise (208). Importantly, home-based and remotely supervised aerobic programs, such as the Park-in-Shape trial, have shown efficacy in reducing motor symptoms, highlighting their practicality and long-term feasibility (209). Overall, a wide range of exercise modalities, from yoga and resistance training to treadmill and high-intensity aerobic interventions, have demonstrated significant benefits for people with Parkinson's disease. These include improved gait, muscle strength, motor performance, cognitive function, and mood, along with enhanced quality of life. Mechanistic studies indicate that these outcomes are mediated through mitochondrial support, antioxidant defense, neuroplasticity, and dopaminergic regulation. Exercise is therefore a powerful, non-pharmacological adjunct in PD management, adaptable to different stages and patient needs.

## 5 Synergistic benefits of saffron and exercise in age-related diseases

**Figure 4:**
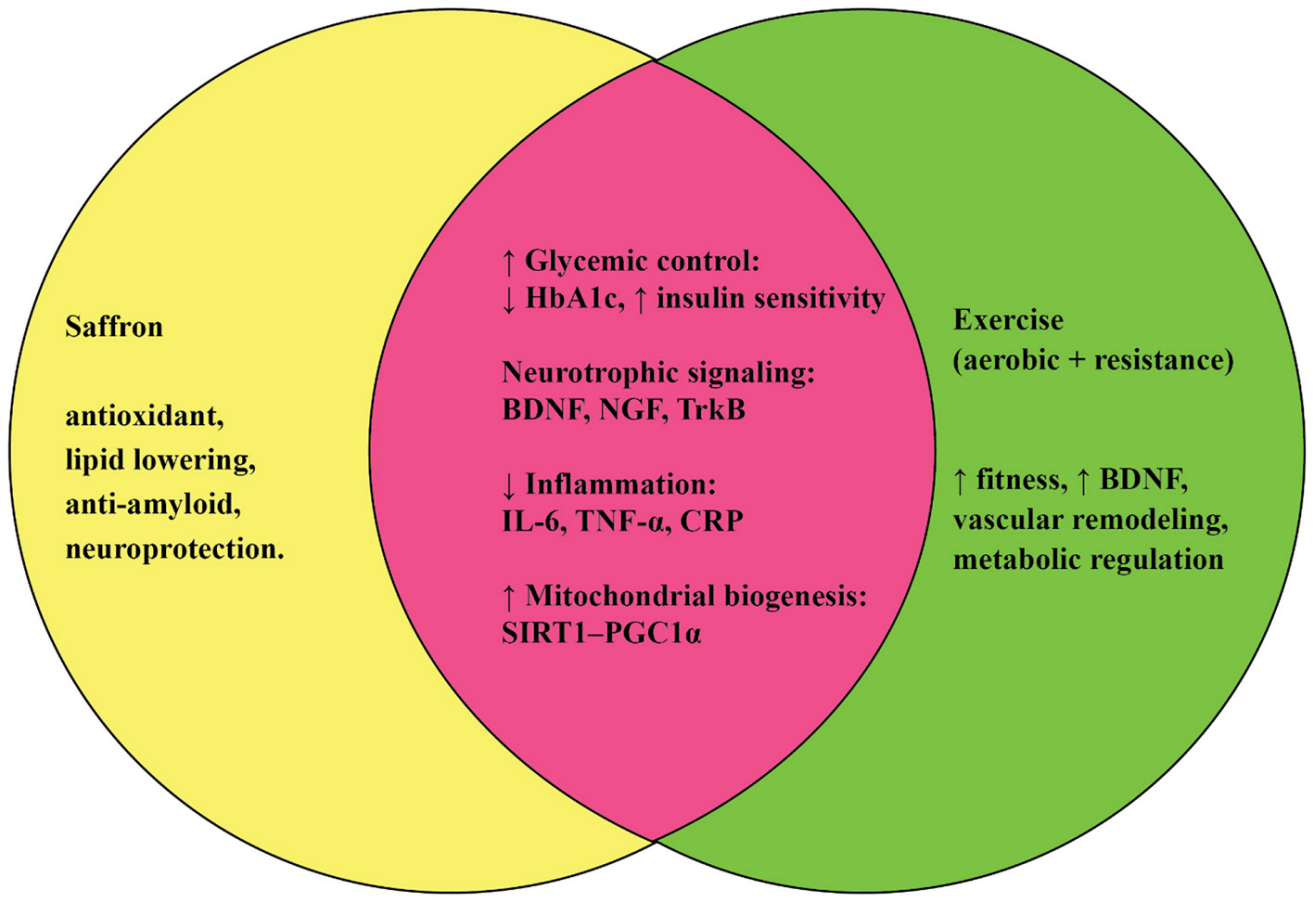
Synergistic pathways of saffron and exercise in healthy aging.

The growing evidence on the convergence of nutraceutical and lifestyle interventions highlights the unique potential of saffron and physical activity as complementary strategies in aging-related disorders. While each intervention independently exerts significant effects on metabolism, vascular health, and neurodegeneration, their combined use produces amplified outcomes across multiple biological systems. Recent human and animal studies consistently indicate that saffron potentiates the effects of exercise on glycemic regulation, vascular remodeling, neurotrophic signaling, and inflammatory suppression, offering a multi-layered defense against the hallmarks of age-related diseases (Figure 4). A summary of the current experimental and clinical evidence on combined saffron–exercise interventions is presented in Table 3, highlighting their synergistic effects across metabolic, cardiovascular, and neurodegenerative outcomes.

**Table 3:** Combined effects of exercise and saffron supplementation on metabolic and neurodegenerative disorders.

**Model**	**Intervention**	**Main findings**	**Mechanisms**	**References**
Diabetic rats	Resistance exercise + saffron extract	↓ glucose, lipids, HbA1c; ↑ GLUT4, AMPK	Improved insulin signaling, antioxidant action	(212)
Obese women with T2DM	Aerobic training ± saffron (12 wks)	Greatest ↓ in HOMA-IR, IL-6, TNF-α in combined group	Anti-inflammatory, insulin sensitization	(213)
Obese men with T2DM	Concurrent training ± saffron (12 wks)	CTS group showed largest ↓ in CRP, IL-1β, HbA1c, and ↑ IL-10	Cytokine modulation	(214)
Obese women with T2DM	Aerobic training ± saffron (8 wks)	↓ body fat, LDL, resistin, TNF-α; ↑ adiponectin, HDL-C	Improved lipid metabolism, reduced inflammation	(215)
T2DM men	Aerobic training ± saffron	↑ ApoA1, ↓ Troponin T, ↓ HFABP with combo	Myocardial protection	(216, 217)
Diabetic rats	Resistance or HIIT + saffron	↓ HSPs, IL-12, TNF-α	Anti-inflammatory, immune modulation	(218)
Elderly hypertensive men	Resistance training ± saffron	↓ SBP, ↑ NO, adiponectin, ↓ ET-1	Endothelial regulation	(210)
Elderly hypertensive men	Resistance training ± saffron	↓ leptin, resistin, MCP-1, IL-6	Reduced vascular inflammation	(211)
AD rats	Endurance training + crocin	↑ memory, NGF, BDNF, TrkB; ↓ tau	Neurotrophic signaling, anti-tau	(219)
AD rats	Endurance training + saffron	↑ hippocampal PGC-1α	Mitochondrial biogenesis	(220)
AD rats	Endurance training + crocin	↑ IGF-1, glycogen	Neurotrophic, metabolic effects	(221)
AD rats	Endurance training + saffron	↑ miR-29a, ↓ depression	Epigenetic regulation	(222)
AD rats	Endurance training + crocin	↓ anxiety, ↑ aerobic power	Behavioral modulation	(223)
PD rats	Treadmill + crocin	Improved motor/memory, ↓ TNF-α, oxidative stress	Antioxidant, anti-inflammatory	(224)

The growing body of mechanistic research reveals that saffron and exercise converge on interconnected signaling cascades, particularly the PI3K/Akt/mTOR, AMPK/SIRT1-PGC-1α, and Nrf2-mediated pathways, that coordinate oxidative balance, mitochondrial biogenesis, and neurotrophic signaling. Activation of these shared molecular targets promotes autophagic clearance of damaged proteins, improves cellular energy homeostasis, and enhances the expression of BDNF. Through these overlapping mechanisms, saffron and exercise reinforce each other's antioxidant and anti-inflammatory actions, providing a biological rationale for their observed synergy across metabolic and neurodegenerative disorders. Conceptually, this framework underscores the novelty of integrating nutraceutical and lifestyle strategies as complementary, interdependent components of a unified adaptive system for promoting healthy aging.

### 5.1 Cardiovascular benefits in hypertensive aging

Hypertension and vascular dysfunction are critical contributors to frailty and mortality in older adults. Studies indicate that saffron and exercise complement one another in improving endothelial health. In a randomized controlled trial with hypertensive older men, resistance training combined with saffron supplementation reduced systolic blood pressure more effectively than either alone, with significant increases in nitric oxide and adiponectin and decreases in endothelin-1 (210). These vascular adaptations suggest improved endothelial relaxation and reduced vasoconstrictor tone. Mojtahedi et al. (211) extended these findings by showing that combined saffron and resistance training produced stronger reductions in leptin, resistin, MCP-1, and IL-6 than either treatment individually. While saffron and exercise each improved lipid profiles by raising HDL and lowering total cholesterol, their combination did not further augment these effects, suggesting that lipid changes may plateau. However, the additive impact on inflammatory cytokines underscores their shared but distinct pathways, where saffron primarily suppresses NF-κB–mediated inflammation and exercise enhances vascular shear stress–driven nitric oxide bioavailability (211).

### 5.2 Metabolic interactions in type 2 diabetes

Diabetes mellitus represents a paradigm where saffron and exercise act synergistically. At the cellular level, saffron enhances insulin secretion and glucose uptake in β-cells and myotubes, mediated by upregulation of GLUT4 and AMPK pathways. Resistance exercise alone activates these same pathways through mechanical and metabolic stress, but their co-activation markedly improves systemic glycemic control. In rodent models, combined saffron and exercise interventions lowered fasting glucose, HbA1c, and serum lipids while increasing antioxidant defenses, demonstrating improvements that exceeded single treatments (212).

Human trials mirror these preclinical findings. In obese women with T2DM, 12 weeks of aerobic training plus saffron supplementation produced the strongest reductions in HOMA-IR, fibrinogen, homocysteine, and pro-inflammatory cytokines compared with exercise or saffron alone (213). Likewise, in obese men, concurrent training with saffron supplementation resulted in significantly greater declines in TNF-α, IL-6, IL-1β, and hs-CRP, with a concomitant rise in IL-10, pointing to an enhanced anti-inflammatory phenotype (214). Importantly, markers of lipid metabolism, including triglycerides and LDL-C, improved most robustly in the combination groups. These findings suggest that saffron not only mirrors the effects of exercise but also magnifies them through redox-mediated and cytokine-modulating pathways. Notably, the metabolic improvements appear sustainable beyond active training. Rajabi et al. (215) observed that women who received saffron plus aerobic training maintained improvements in insulin sensitivity, adiponectin, and lipid profile for up to 2 weeks after detraining, whereas benefits faded more quickly in groups without saffron. This indicates that saffron may stabilize or prolong the adaptive metabolic response induced by exercise, perhaps through its capacity to buffer oxidative stress and regulate adipocytokines (215).

Beyond glucose control, combined saffron and exercise improves cardiac risk markers. Barari et al. (216, 217) reported that men with diabetes who underwent aerobic training while receiving saffron extract showed significant reductions in ApoA1, Troponin T, and HFABP, suggesting myocardial protection. Given that Troponin T and HFABP are sensitive markers of subclinical cardiac injury, these results emphasize the cardioprotective value of integrating saffron into exercise programs for high-risk patients (216, 217). Animal data further reinforce this conclusion. In diabetic rats, resistance training or HIIT combined with saffron reduced expression of inflammatory mediators such as HSPs, IL-12, and TNF-α (218). Together, these outcomes highlight a coordinated effect on glucose homeostasis, lipid metabolism, and systemic inflammation.

### 5.3 Neuroprotective interactions in Alzheimer's disease

The role of saffron and exercise in neurodegeneration is particularly evident in Alzheimer's disease (AD). In rodent models, both interventions have been shown to influence neurotrophic pathways, tau phosphorylation, and mitochondrial quality control. Moghadasi et al. (219) demonstrated that crocin supplementation combined with endurance training significantly improved memory and learning in TMT-induced AD rats. These effects were associated with the upregulation of BDNF, NGF, and TrkB expression and reduced tau, highlighting enhanced synaptic plasticity and attenuated pathological burden (219). Mitochondrial biogenesis is another area of convergence. Azarian et al. (220) found that endurance training with saffron increased hippocampal expression of PGC-1α, a master regulator of mitochondrial metabolism, beyond the effects of either alone (220). Similarly, Negarandeh et al. (221) showed that endurance training combined with crocin increased IGF-1 and glycogen expression in hippocampal tissue of AD rats, suggesting improved energy supply for neuronal survival. These findings underscore how saffron stabilizes neuronal energy metabolism while exercise drives structural and synaptic adaptations (221).

Psychological dimensions of AD also appear responsive. Negarandeh et al. (222) reported that the combination of endurance training and saffron increased miR-29a expression and produced greater reductions in depression-like behaviors compared with either treatment alone. Another study found that while crocin and exercise independently reduced anxiety and improved aerobic power, their combination did not produce additive effects in these domains (223). Nevertheless, the overall evidence indicates a clear synergy in cognitive and mood-related outcomes.

### 5.4 Interactions in Parkinson's disease

Parkinson's disease (PD) is characterized by dopaminergic degeneration and oxidative injury, both of which are targeted by saffron and exercise. Shahidani et al. (224) investigated treadmill training with crocin in 6-OHDA-lesioned rats, a standard PD model. The combined intervention reduced motor asymmetry, improved spatial and aversive memory, and significantly decreased TNF-α and lipid peroxidation. Biochemical assays showed that crocin enhanced antioxidant capacity, while exercise increased thiol concentration, together producing robust neuroprotection. These findings illustrate that saffron amplifies the neurobiological gains of exercise by directly scavenging free radicals and modulating inflammatory cascades, whereas exercise reinforces synaptic and motor plasticity.

Together, these studies point toward a consistent theme: saffron reinforces the biochemical and cellular adaptations induced by exercise, while exercise facilitates the functional expression of saffron's molecular effects. Their convergence on mitochondrial function, antioxidant defense, and inflammatory regulation explains their broad utility across metabolic, cardiovascular, and neurodegenerative diseases of aging. Thus, accumulating preclinical and clinical data demonstrate that saffron and exercise are not merely additive but often synergistic in combating age-related disease. They converge on AMPK/GLUT4 signaling, nitric oxide production, neurotrophic factor expression, and suppression of inflammatory cytokines, while addressing psychological and functional domains such as depression, fatigue, and mobility. These integrative effects provide compelling evidence that combining saffron supplementation with structured exercise represents a safe, culturally adaptable, and mechanistically grounded strategy to promote health span in older adults.

## 6 Limitations

Although the evidence supporting saffron and exercise as complementary interventions in age-related diseases is steadily growing, several limitations constrain the strength of current conclusions. First, most clinical studies remain small in sample size, short in duration, and often single-centered, which reduces their generalizability to broader populations. Differences in trial design, participant characteristics (age, comorbidities, and baseline disease severity), and outcome measures further complicate cross-study comparisons. Second, saffron preparations are heterogeneous, with variable dosages, extraction methods, and purity across studies. This lack of standardization makes it difficult to establish optimal dosing regimens or to compare results across trials. Moreover, many studies do not perform chemical characterization of saffron supplements, leaving uncertainty about the precise concentrations of crocin, crocetin, and safranal being administered.

Third, while animal models provide mechanistic insight, they cannot fully replicate the multifactorial complexity of human aging. Rodent models of Alzheimer's, Parkinson's, or diabetes capture only selected aspects of human disease, which limits the translation of preclinical findings to clinical practice. Fourth, most human trials assess short-term biochemical or functional endpoints (e.g., insulin resistance, inflammatory markers, cognition over 12–24 weeks), but long-term data on clinical outcomes such as cardiovascular events, disease progression, or mortality are lacking. Additionally, adherence to both exercise and supplementation protocols is rarely monitored beyond the intervention period, leaving the sustainability of benefits unclear. Finally, the interaction between saffron and exercise at the molecular level remains incompletely understood. While both converge on pathways such as AMPK, NF-κB, and BDNF signaling, the precise temporal sequence and dose–response relationships are not well-defined. This gap makes it difficult to determine whether saffron enhances exercise adaptations directly or whether its benefits arise mainly from independent parallel mechanisms.

## 7 Future perspectives

Future work should prioritize large-scale, multicenter randomized controlled trials that investigate the combined effects of saffron supplementation and structured exercise programs in diverse populations. Standardization of saffron formulations is essential, with precise quantification of active compounds such as crocin and crocetin to ensure reproducibility. Dose–response studies are particularly needed to determine the minimal effective and maximal safe doses when combined with exercise. Longitudinal research should assess not only surrogate markers (e.g., HbA1c, inflammatory cytokines, and neurotrophins) but also hard clinical outcomes, including incidence of dementia, cardiovascular events, functional disability, and quality-adjusted life years. Extending follow-up beyond the typical 8–24 weeks will help establish the durability of benefits and adherence patterns.

Mechanistic studies should focus on dissecting how saffron influences exercise-induced adaptations in mitochondria, synaptic plasticity, endothelial function, and immune regulation. Integrative omics approaches (transcriptomics, metabolomics, and proteomics) may uncover novel pathways and biomarkers that reflect the synergy between nutraceuticals and lifestyle interventions. Another priority is personalization. Not all individuals respond equally to exercise or saffron, and genetic, metabolic, and microbiome profiles may influence outcomes. Precision-nutrition and precision-exercise frameworks could help tailor interventions to maximize benefit for specific subgroups, such as postmenopausal women, patients with metabolic syndrome, or individuals at high risk for neurodegeneration. In alignment with the latest advances, recent comprehensive reviews have broadened the therapeutic scope of saffron beyond metabolic and neurodegenerative contexts. Razavi et al. (225) demonstrated that saffron and its key constituents, crocin, crocetin, safranal, and kaempferol, ameliorate oxidative stress, inflammation, and apoptosis in models of traumatic brain injury via modulation of NF-κB, NLRP3, Nrf2/HO-1, Bcl-2/Bax, and SIRT1 signaling. These mechanistic pathways parallel those activated by exercise-induced neuroplastic and mitochondrial adaptations, supporting the broader neuroprotective relevance of saffron. Likewise, Goyal et al. (226) summarized saffron's benefits in reproductive and sexual health, including improved erectile and menstrual function through serotonergic, nitric-oxide–dependent, and anti-inflammatory mechanisms. Together, these findings highlight saffron's multifaceted physiological influence across neuroendocrine and systemic domains, reinforcing its potential as a holistic adjunct to lifestyle-based interventions such as structured physical activity. Building upon these recent insights, future research should explore how saffron's multi-systemic effects can be harnessed synergistically with exercise training, for example, by integrating neuroprotective, endocrine, and vascular endpoints into combined intervention trials. Such cross-domain designs could clarify whether saffron enhances exercise-mediated adaptations in cognitive, reproductive, and metabolic health, thereby providing a mechanistic foundation for personalized lifestyle–nutraceutical strategies across the lifespan.

In addition to scientific and mechanistic advances, the integration of saffron supplementation and structured exercise also carries important global-health and policy implications. Within the framework of the WHO Decade of Healthy Ageing (2021–2030), such strategies align with international priorities to promote functional ability, equity, and active participation of older adults. Both saffron and exercise represent safe, low-cost, and culturally adaptable interventions that can be implemented across diverse socioeconomic settings. Their accessibility is particularly relevant for low- and middle-income countries, where pharmaceutical options for chronic disease prevention are limited but community-based health programs are expanding. Future interdisciplinary collaborations between clinicians, public-health professionals, and policymakers will be crucial to translate this nutraceutical-lifestyle synergy into scalable, sustainable models for healthy aging worldwide. Finally, pragmatic trials and implementation studies are needed to evaluate the feasibility, cultural adaptability, and cost-effectiveness of combined saffron–exercise programs in real-world settings. This is particularly relevant in low- and middle-income countries, where saffron is culturally accepted and exercise interventions are cost-efficient but underutilized.

## 8 Conclusion

The current body of evidence underscores saffron and exercise as two promising non-pharmacological interventions with complementary effects on age-related diseases. Saffron and its bioactive components modulate oxidative stress, inflammation, mitochondrial dysfunction, and aberrant protein aggregation, while exercise improves cardiovascular fitness, metabolic health, neuronal resilience, and psychological wellbeing. When combined, these interventions appear to exert synergistic benefits, enhancing glycemic control, vascular integrity, neurotrophic signaling, and functional outcomes across experimental and clinical models. Although encouraging results support their potential as low-risk adjunct therapies, existing clinical studies remain limited in size, duration, and methodological consistency. Future well-designed trials are essential to confirm the long-term efficacy, clarify optimal dosing and exercise regimens, and identify patient populations most likely to benefit. Collectively, saffron and exercise represent complementary and biologically active strategies with significant potential to improve healthspan and mitigate the burden of age-related diseases.
